# Consumer Disposition Toward Fairness in Agri-Food Chains (FAIRFOOD): Scale Development and Validation

**DOI:** 10.1007/s10551-024-05756-2

**Published:** 2024-07-19

**Authors:** Margherita Del Prete, Artyom Golossenko, Matthew Gorton, Barbara Tocco, Antonella Samoggia

**Affiliations:** 1 Alma Mater Studiorum, Department of Agricultural and Food Sciences, University of Bologna, Bologna, Italy; 2 Cardiff Business School, Cardiff University, Cardiff, Wales UK; 3 Newcastle University Business School, Newcastle University, Newcastle Upon Tyne, UK; 4 Corvinus University of Budapest, Fővám Tér 8, Budapest, 1093 Hungary; 5 National Innovation Centre for Rural Enterprise, Newcastle University, Newcastle Upon Tyne, UK

**Keywords:** Fairness, Agri-food supply chain, Scale development

## Abstract

Fairness in agri-food supply chains receives increasing consumer, industry, and political attention but is currently under-conceptualized and lacks appropriate frameworks for measurement. Therefore, building on a theoretically grounded conceptualization of consumer dispositions toward fairness in agri-food supply chains, we developed and validated a 14-item fairness measurement scale (FAIRFOOD). The scale comprises of four dimensions (economic, environmental, social, and informational) which are manifestations of the same construct (higher-order structure). We empirically validate the scale and its reliability using four studies and eight independent samples from Italy (*n* = 1386) and the UK (*n* = 1379). The findings reveal that FAIRFOOD is related, yet distinct from theoretically relevant constructs such as ethical consumption and pro-environment behavior. The FAIRFOOD scale is a strong predictor of outcomes such as willingness to purchase Fairtrade certified products, as well as boycott and negative Word of Mouth intentions if a brand treats other supply chain actors unfairly. Regarding business strategy, rather than focusing on one dimension of fairness independently, managers should adopt a holistic approach, devising initiatives that address all four dimensions in tandem.

**Supplementary Information:**

The online version contains supplementary material available at 10.1007/s10551-024-05756-2.

## Introduction

What makes an action, situation, or outcome fair or unfair has long been a central question in moral philosophy (Rawls, 1973). However, fairness in agri-food supply chains has only recently received sustained attention from scientific and policy communities (Busch & Spiller, 2016; Samoggia et al., 2021). Agri-food chain fairness matters because fair compensation for farmers and fair pricing for consumers contribute to economic equity, provide a basis for more sustainable farming practices, and responsible resource management, influencing the availability of affordable, nutritious and safe food (European Commission, 2016, 2021). While fairness is increasingly considered across different supply chains, it is particularly critical in the agri-food sector for three main reasons. First, disparities in economic size and power are especially large in agri-food supply chains, for instance between small-scale producers in emerging economies, as well as upland farmers in developed countries, on the one hand, and large, multi-national food processors and retailers on the other (Hingley, 2005). Second, because agriculture accounts for one-half of all the world’s habitable land, and agri-food systems contribute one-third of all greenhouse gas emissions, agri-food supply chains are most prominent in debates concerning the transition to more responsible production and consumption systems (FAO, 2019, 2022). Thirdly, political initiatives to ensure fairness in supply chains are more widespread and pronounced in the case of the agri-food sector. For instance, the European Union’s flagship “Farm to Fork” strategy seeks to build ‘fair, strong and sustainable food systems’ (European Commission, 2020, p. 12), which builds on legislation designed to reduce the incidence of unfair trading practices in agri-food supply chains (European Commission, 2021). Elsewhere, states have also established regulatory bodies and/or introduced laws (HM Government, 2013; The White House, 2022) intended to ensure that agri-food supply chains deliver fairer outcomes for producers, in response to perceived imbalances in power between supply chain actors, concerns regarding producer remuneration, and political actions such as farmers’ strikes and protests (Busch & Spiller, 2016; Falkowski et al., 2017; Swinnen et al., 2021). In addition to government actions, a plethora of non-government organizations and commercial entities introduced initiatives designed to establish fairer agri-food supply chains (Goossens et al., 2017; Gorton et al., 2023). Initially, these attempts tended to focus either on environmental justice concerns, or socio-economic outcomes as embedded in Fair Trade (FT) certification. More recently, both academic and practitioner interests have shifted to holistic, ‘triple bottom line’ (profit, people, planet) approaches (Manika et al., 2015; Senyo & Osabutey, 2021) in support of supply chains that are fairer economically, socially, and environmentally, with transparent outcomes communicated to end users (Bellassen et al., 2022; Cadden et al., 2015; Gregory-Smith et al., 2017; von Berlepsch et al., 2022). This holistic approach recognizes that attempts to improve producers’ returns that are, for example, environmentally damaging are undesirable in the long run. Similarly, initiatives to create more environmentally friendly supply chains will fail if they are economically and socially unsustainable, due to a lack of lack financial viability and political support. Consequently, consumer engagement is a necessary condition for sustaining fairer agri-food supply chains (Kutaula et al., 2022).

To date, the literature lacks a scale to measure consumers’ dispositions toward fairness in agri-food supply chains. There are studies that explore interest, beliefs, perceptions, and willingness to buy specific products such as FT certified goods (Alzubaidi et al., 2021; De Pelsmacker & Janssens, 2007; Kilbourne & Pickett, 2008; Shih-Tse Wang & Chen, 2019; Toti et al., 2021). Yet, studies that focus on consumers' attitudes and behaviors related to products that carry FT certification lack a holistic coverage of the dimensions of fairness, because they fail to integrate consideration of economic, social, and environmental aspects. Similarly, measures of Ethically Minded Consumer Behavior (EMCB) (Sudbury-Riley & Kohlbacher, 2016) focus mainly on environmental and corporate social responsibility aspects, omitting consideration of transparency and fair returns to producers. Consequently, there is a mismatch between the triple bottom line approach to fairness, which increasingly informs research and policy, and the tools available to capture individual-level differences in consumers’ dispositions to this agenda. The objective of this paper thus is *to conceptualize and develop a valid and reliable scale to measure consumers’ dispositions toward fairness in agri-food supply chains*.

The paper makes three main contributions. Firstly, drawing on Fairness Theory (Broome, 1990; Folger & Cropanzano, 2001), it defines and provides a multidimensional conceptualization of consumers’ dispositions toward fairness in agri-food chains that encompasses the key attributes of the construct. Second, based on this novel conceptualization, the study offers a reliable, valid, and invariant multidimensional measurement of consumers’ dispositions toward fairness in agri-food chains across two countries—Italy and the United Kingdom (UK). Specifically, the research proposes a four-dimensional scale of consumers’ dispositions toward fairness in agri-food supply chains (labelled here as FAIRFOOD), providing a necessary and critical tool to advance a more comprehensive conceptualization of the nomological network of the construct. Finally, we provide evidence of the predictive validity of the proposed construct for both purchase and brand activism related outcomes, enhancing understanding of how consumers’ dispositions toward fairness affect their commitment, engagement, and emotional experiences in food decisions.

For scale development, we selected Italy and the UK to represent two contrasting food cultures. Italy has largely retained its regional food specialties, is a world leader in the number of protected geographical indications (AND-International, ECORYS, & COGEA, 2020), pioneered the development of solidarity purchasing groups between producers and consumers, and had over 1.1 million farms in 2020 (Eurostat, 2022). Most farms in Italy are small by international standards, with a mean size of 11 hectares (CREA, 2022), and they are often exposed to unfair food chain practices compensated by consumers’ purchasing practices (Fonte & Cucco, 2017). In contrast, the UK’s food system is more concentrated, based on manufacturer and retailer brands, larger farms, and less regional differentiation (Blundel & Tregear, 2006). There are now only around 216,000 farms in England, with an average farm size of 85 hectares (Defra, 2022)—over seven times larger than Italy. The UK also relies on imported food products based on trade agreements which seek to provide consumers with food respecting ethical standards in the global food system (Trade & Agriculture Commission, 2021).

The remainder of the article is structured as follows. Firstly, the paper defines and conceptualizes consumers dispositions toward fairness in agri-food supply chains, summarizing each dimension of the concept. Secondly, following contemporary, accepted procedures for scale development (DeVellis, 2016) we develop and validate a four-dimensional second-order measurement scale using subject-matter experts and four independent samples of consumers in Italy and the UK. As part of the process, we detail how we generated and reduced the initial pool of items and assessed psychometric properties (i.e., factor structure, measurement, invariance, reliability, convergent, discriminant validity) and the nomological network of the measurement. Finally, we discuss the theoretical and managerial implications of our scale development and predictive relevance.

## Background

### Theoretical Background

Fairness is a moral disposition (Fowers et al., 2022) that explains cross-situational tendencies regarding what people regard as just outcomes, situations and events (Colquitt et al., 2018). As a disposition, fairness affects attitude and behavior (Colquitt et al., 2001), and is influenced by moral identities and personality traits (McFerran et al., 2010). Consequently, fairness, as a quality of character, will vary substantially across individuals (Colquitt et al., 2018; Gamliel et al., 2014), helping explain, differences in reactions to relevant stimuli such as FT products or whether to boycott or bad mouth actors perceived to act unfairly.

Fairness theory suggests that fairness has three interrelated components (Folger & Cropanzano, 2001), which Broome, (1990) labels as: teleological claims regarding the nature and distribution of outcomes; rights including constraints on the behavior of actors; and duties and obligations to others. Within the context of agri-food supply chains, it thus concerns normative judgments regarding the outcomes and distribution of benefits, the rights of, and constraints on, actors within the supply chain, and actors’ duties and obligations to others including the environment (Busch & Spiller, 2016). In this context, we define *fairness as the degree to which an individual regards it as important that other actors in the agri-food supply chain are treated fairly, concerning the outcomes and distribution of benefits, rights and constraints on different actors, and the duties and obligations to others*. This broad and comprehensive interpretation, consistent with Fairness Theory, provided an initial conceptualization of fairness, from which the items for the development of the scale were then extracted.

In keeping with Fairness Theory (Broome, 1990; Folger & Cropanzano, 2001), the definition of fairness captures its multi-dimensionality. Outcomes relate to distributive justice and how returns are allocated between supply chain actors—the economic or ‘profit’ element of the triple bottom line (Senyo & Osabutey, 2021). Rights concern the protection of individuals from exploitation, fair remuneration, and safe working conditions (social fairness)—the ‘people’ element in the triple bottom line. Obligations to others including the environment involve duties to future generations and other species (environmental fairness)—in other words, the ‘planet’ element of the triple bottom line. For consumers to make choices consistent with their dispositions requires relevant and truthful information (informational fairness). We thus conceptualize FAIRFOOD as having four dimensions—economic fairness, social fairness, environmental fairness, and informational fairness. The rest of this section outlines each dimension and previous measures. Reviewing extant research reveals the absence of an existing scale to measure FAIRFOOD or something closely resembling it.

#### Economic Fairness

Economic fairness refers to the distribution of profits between agri-food supply chain actors. Distributive fairness has its origins in the equity theory of Adams, (1963), based on the concept that the outcome, interpreted as the returns each actor receives for its products, is fair if it allows actors along the chain to make reasonable and proportionate profits (Busch & Spiller, 2016; Gudbrandsdottir et al., 2021). Economic fairness is often seen from the point of view of farmers, who are considered the most vulnerable and disadvantaged actor in agri-food supply chains (Samoggia et al., 2021). However, economic fairness should be conceptualized as fair distribution among all supply chain stakeholders, in terms of value captured (Briggeman & Lusk, 2011). Some legislation strives for economic fairness—for instance the EU Directive on Unfair Trading Practices in Business-to-business Relationships in the Agricultural and Food Supply Chain (European Commission, 2021) seeks to promote fairer economic outcomes by restricting the exploitation of power imbalances. Consumers may interpret economic fairness in terms of the price they pay for products (Bolton et al., 2003; Diller, 2008) as well as the returns to other actors in the supply chain, especially farmers. The principle of economic fairness is at the core of the FT movement, and several studies develop measures of consumers’ attitude to FT and the perceived distributive justice of Fair-Trade Organizations (FTOs) (Shih-Tse Wang & Chen, 2019), analyzing their effect on food purchase intentions.

#### Social Fairness

Social fairness relates to the protection of human rights, safe and healthy working conditions, free choice of employment, and fair remuneration for one’s work (Nickel, 2023). It thus incorporates notions of interpersonal fairness (Greenberg, 1990)—treating people with dignity and respect. A supply chain perspective is integral to the enactment of social fairness in law. For instance, the proposed EU Directive on Mandatory Human Rights, Environmental and Good Governance Due Diligence requires that companies falling within its scope must identify their suppliers and subcontractors and implement actions in accordance with the company’s due diligence strategy. This includes measures relating to occupational health and safety, working hours and workload, labor exploitation including child labor, as well as the sustainable use of natural resources (European Parliament, 2021). In addition to public regulation, many grocery retailers enforce codes of conduct, designed to be consistent with the International Labour Organization (ILO) core conventions, on their agri-food suppliers. This is partly to protect the retailers’ reputations as unfair treatment of workers in their supply chains may promote consumer backlashes and boycotts (Tian et al., 2021). While scales to measure the importance to consumers of social fairness in supply chains are limited, many studies measure consumer interest in FT certification (De Pelsmacker & Janssens, 2007; Shih-Tse Wang & Chen, 2019). In addition, Toti et al., (2021) addressed the topic of child labor and employee rights and Sudbury-Riley & Kohlbacher, (2016) included items in their EMCB scale that consider the influence of food companies' social responsibility in ensuring safe working conditions and avoiding labor exploitation on consumers' purchasing behavior.

#### Environmental Fairness

Environmental fairness refers to the remediation of existing, and prevention of future, ecological damage (Abramovich & Vasiliu, 2022), in ways consistent with the definition of sustainability of the Brundtland Commission, (1987)—meeting the needs of the present without compromising the ability of future generations to meet their own needs. The latter acknowledges that a fair distribution of environmental resources between present and future generations is an integral dimension of fairness. This principle underpins actions on climate change and efforts to reduce the ecological damage of agri-food supply chain practices (European Commission, 2020).

Environmental fairness thus exists at the nexus of food production and consumption, underpinned by principles concerning the primacy of ecological preservation and human welfare (Abramovich & Vasiliu, 2022; Fairtrade International, 2023; FAO & OECD, 2023). Central to environmental fairness is the imperative to safeguard the inherent integrity of the food environment, including soil conservation, biodiversity protection, water and waste management as well as the promotion of health and safety standards, alongside the conscientious treatment of animals (FAO & OECD, 2023; Food Ethics Council, 2020). As a practical manifestation, organic food seeks to ensure sustainable agricultural practices and environmental fairness (Lusk & Briggeman, 2009; Toti et al., 2021).

Environmental fairness is more important to some consumers than others, and this affects their purchasing behavior—for instance it helps explain the likelihood of purchasing organic food and willingness to pay for sustainable packaging (Golob et al., 2018; Thφgersen, 1999). There are various scales measuring consumers’ environmental consciousness. For instance, Kilbourne and Pickett, (2008) developed a scale that measures environmental beliefs, concerns, and behaviors, while Alzubaidi et al., (2021) examine the antecedents of consumers’ pro-environmental behavior. Toti et al., (2021) study the effect of ethical sensitivity on consumers’ interest in eco-labelled products. However, most extant research analyzes environmental fairness in isolation, rather than regarding it as one dimension of a fairness disposition. Uniquely, Sudbury-Riley & Kohlbacher, (2016) developed and validated an EMCB scale which encompasses issues like reusable and recycled packaging. However, none of the mentioned studies address specific environmental aspects of supply chains but rather environmental issues more generally.

#### Informational Fairness

Informational fairness concerns the quality and quantity of information shared with consumers and between other actors in the supply chain (Greenberg, 1990). Sharing information fairly implies communicating truthfully with other actors, so that in exchange relationships buyers and sellers can make decisions consistent with their preferences. A fundamental tenet of a just system is that stakeholders are able to take informed actions and give informed consent (Food Ethics Council, 2020). The ability to take informed actions or give informed consent is constrained when relevant information is withheld or misconstrued, especially in situations where buyers cannot directly and easily verify the claims made. For instance, ‘greenwashing’ whereby sellers make false or misleading claims regarding the environmental benefits of a good or service (van der Ven, 2019), contravenes the principles of information fairness.

Informational fairness underpins European food labelling regulations, which seek to enhance information and transparency in supply chains (European Parliament, 2011). Regulations acknowledge that the absence of informational fairness may lead to market failure, where consumers cannot accurately and easily distinguish truthful from false claims, as well as a loss of trust. ‘Fairer’ products often involve higher costs and their viability in a market typically depends on buyers being willing to pay a premium for them (Dammert & Mohan, 2014). However, buyers are less likely to pay a premium in environments where they lack trust in sellers, regulatory institutions, and the information provided to them (Gorton et al., 2021). Consequently, the purchase of products which support fairer supply chain outcomes depends on informational fairness, as acknowledged in the FT buying behavior model developed by De Pelsmacker & Janssens, (2007), where information about FT is an antecedent of attitudes to FT and buyer behavior.

### FAIRFOOD: Multidimensionality and Second-Order Structure

Based on the above conceptualization, we envision that FAIRFOOD is a superordinate (second-order), multidimensional construct. This reflects that the four dimensions are different manifestations of the same underlying construct of fairness which represents the commonality between the dimensions (Edwards, 2001; Law et al., 1998). The multidimensional construct thus cannot be conceived separately from its specific dimensions. Consequently, we construe the second-order latent factor as an abstract and embedded representation of an overall disposition towards fairness, whereas the four dimensions constitute less abstract, specific components of fairness in the form of economic, social, environmental, and informational dimensions. Furthermore, specifying the multidimensional construct of fairness as a second-order allows us to conduct analyses at the construct level since we seek to draw conclusions about the overall multidimensional construct instead of its individual dimensions (Edwards, 2001; Wong et al., 2008).

## Study 1: Content Validity and Latent Structure

The objectives of this study were to (1) generate a set of items that constitute the concept of fairness in agri-food supply chains, (2) examine the face validity of the initial set of items, (3) examine the factorial composition of the generated items using Exploratory Structural Equation Modelling (ESEM), (4) retain a parsimonious set of items, and (5) conduct an initial psychometric assessment of the retained items.

### Item Generation and Content Validation

Following the procedures for scale development detailed in DeVellis, (2016), we generated a broad set of items to capture the potential aspects of fairness in agri-food supply chains, drawing on a comprehensive literature review. Based on the preceding conceptualization of fairness, we included topics related to the concepts of sustainability, social justice, and equity in agri-food supply chains, as well as the academic literature that explores consumer interest in products that ensure fairness within agri-food supply chains. Based on this literature review, we identified an initial pool of 42 items which encompassed different aspects of fairness in agri-food supply chains (Appendix Table 1).

Next, we solicited content validity ratings on this pool of items from twelve experts familiar with the subject matter. We included academics and experts within fairness-related organizations, both from Italy and the UK. Following the approach of Zaichkowsky, (1985), we provided experts with a definition of fairness and asked them to rate each item with respect to its relevance to the definition—‘low’, ‘moderate’ or ‘high’. We retained items if they were rated as (i) ‘high’ by more than 50% of the experts or (ii) ‘moderate’ or ‘high’ by at least 80% of the experts. Experts also provided comments on the items’ ambiguity, clarity, and redundancy as well as suggestions to consolidate item wording. We reviewed the qualitative feedback for possible item revisions (DeVellis, 2016). This process resulted in a pool of 28 items.

To further increase content and face validity, we subjected the items to two sorting exercises. Using respondents’ level of agreement, we attributed each item to a specific dimension (e.g., social, economic). In the first sorting task, a sample of 27 UK participants, recruited via the online research platform Prolific, read a short definition of the different dimensions of fairness and then organized the items by category, as they deemed appropriate. The ‘not belonging to any group’ option was added in case some items did not fit into any dimension. Fifteen items reached more than 70% consensus on belonging to the same dimension. To further increase domain and face validity, we administered a second exercise with a different sample of 27 UK participants who assessed remaining/reworded items. At the end of the process, we retained 20 items, of which four items measured the social dimension, six the economic dimension, four the informational dimension, and six the environmental dimension.

Since our aim was to develop a scale applicable in both Italy and the UK, without restrictions to a particular culture, we also undertook a translation process to avoid language issues. Following Brislin, (1970), the instrument was translated into Italian and translated back to English to avoid errors that can lead to different meanings across the two countries. The translation was undertaken by two independent Italian researchers fluent in English, and external to the study.

### Participants and Procedure

Following previous recommendations (e.g., Worthington & Whittaker, 2006), in this and subsequent studies we considered a sample size of 300 as sufficient for accurate parameter estimations during covariance-based SEM. We collected data from adults in Italy and the UK using Prolific. A total of 423 adults (43.5% female; 54.85% male) agreed to participate in Italy. Most participants reported an age between 18–24 (41.13%) and 25–34 (40.43%), followed by 35–54 (15.84%) and 55–65 (2.60%). In the UK, we recruited 321 adults (54.52% female; 43.93% male). Predominantly participants were aged 35–54 (32.71%), followed by 25–34 (24.30%), 55–65 (19.94%), 18–24 (13.4%), and over 65 (9.66%). Participants were asked to what degree they agreed with each of the 20 FAIRFOOD items was important to them in their food choices on a 7-point fully labelled Likert scale (1 = strongly disagree, 7 = strongly agree), followed by a socio-demographic survey. Italian and English language versions of the questionnaire were prepared, following the procedures discussed above.

### Analysis and Results

#### Assessment of the Factorial Structure

Prior the assessment of the factorial structure of the measurement, we computed the Kaiser–Meyer–Olkin (KMO) measure of sampling adequacy. The results showed that the data were appropriate for further analysis (Italy: KMO = 0.93; UK: KMO = 0.95). We first examined the number of factors using Horn’s parallel analysis. For both the Italian and UK samples, the results of the parallel analysis demonstrated that only the first four eigenvalues were greater than the comparison eigenvalues (using both the mean and 95th percentile criteria) generated by the parallel analysis, therefore indicating four factors should be retained (Hayton et al., 2004). The Velicer MAP criterion and Bayesian Information Criterion (BIC) indicated the extraction of three and four factors respectively for the UK sample. For the Italian sample, both tests suggested the extraction of four factors. Based on the results, we specified a four-factorial solution in subsequent analysis.

We then conducted ESEM using robust Maximum Likelihood Estimation (MLR) with oblimin rotation. ESEM incorporates the strengths of a Confirmatory Factor Analysis (CFA) and Exploratory Factor Analysis (EFA) within a SEM framework (Asparouhov & Muthén, 2009). Consistent with EFA, in ESEM all indicators are permitted to load on all factors, allowing for free estimation of all cross-loadings. Regarding consistency with CFA, ESEM provides a robust means of evaluating model adequacy (e.g., standard errors for parameter estimates and goodness-of-fit indexes). Across studies, in evaluating model fit we used combinations of multiple goodness-of-fit indexes and conventional evaluative criteria (e.g., Hu & Bentler, 1999): comparative fit index (CFI ≥ 0.90 or > 0.95), Tucker–Lewis’s index (TLI ≥ 0.90 or > 0.95), root mean square error of approximation (RMSEA ≤ 0.08 or ≤ 0.10) and the standardized root mean squared residual (SRMR ≤ 0.08).

The four-factorial exploratory model with 20 items demonstrated a good fit to the data for the Italian (*χ*^2^ (116) = 197.42, CFI = 0.98, TLI = 0.97, RMSEA = 0.04, SRMR = 0.02) and the UK (*χ*^2^ (116) = 187.91, CFI = 0.98, TLI = 0.97, RMSEA = 0.04, SRMR = 0.02) samples. Next, to retain the items that most clearly represent the underlying construct, we iterated ESEMs considering the criteria for item retention. Specifically, we removed items that (1) loaded lower than 0.50 on the intended factor or (2) cross-loaded on any other factor at 0.25 or greater (e.g., Tabachnick & Fidell, 2013). We conducted the item removal process simultaneously for both Italy and UK. In each iteration, we removed items when they met the exclusion criteria for at least one group, and we performed a new ESEM within each sample every time we removed an item. In each iteration we also performed parallel analysis to ensure that the item removal did not distort the factorial structure. Through the iterative process, we excluded six items. Table 1 summarizes the results. The remaining 14 items (see Table 2) loaded significantly and substantially on their primary factors (λ > 0.50, all *p* < 0.001) with insubstantial cross-loadings on other factors. The first factor was interpreted as social (3 items), followed by economic (4 items), informational (3 items) and environmental (4 items) fairness.

**Table 1:** ESEM results and psychometric properties of FAIRFOOD (Study 1)

Item	Italy				UK			
	1	2	3	4	1	2	3	4
FAIRFOOD 2	**0.91 (0.94)**	0.05	0.00	− 0.01	**0.85 (0.94)**	0.04	0.06	0.04
FAIRFOOD 3	**0.90 (0.87)**	− 0.06	− 0.01	0.04	**0.98 (0.88)**	− 0.06	− 0.04	0.00
FAIRFOOD 4	**0.85 (0.90)**	0.06	0.03	− 0.01	**0.72 (0.92)**	0.20	0.04	0.03
FAIRFOOD 5	0.07	**0.67 (0.71)**	0.05	− 0.05	0.12	**0.51 (0.77)**	0.23	0.01
FAIRFOOD 7	0.04	**0.59 (0.70)**	0.16	0.00	0.04	**0.67 (0.85)**	0.08	0.15
FAIRFOOD 8	0.01	**0.89 (0.90)**	− 0.04	0.05	− 0.03	**1.03 (0.95)**	− 0.04	0.00
FAIRFOOD 9	0.01	**0.84 (0.85)**	0.00	0.03	0.16	**0.75 (0.95)**	0.08	0.01
FAIRFOOD 11	− 0.04	0.07	**0.82 (0.84)**	0.02	− 0.03	0.04	**0.92 (0.92)**	− 0.01
FAIRFOOD 12	0.09	− 0.09	**0.88 (0.90)**	0.06	− 0.01	0.03	**0.88 (0.93)**	0.06
FAIRFOOD 13	− 0.04	0.06	**0.83 (0.81)**	− 0.06	0.05	− 0.06	**0.88(0.85)**	− 0.03
FAIRFOOD 16	− 0.09	− 0.02	0.02	**0.86 (0.79)**	− 0.02	0.10	− 0.04	**0.81 (0.83)**
FAIRFOOD 17	0.05	0.02	0.00	**0.88 (0.93)**	0.02	− 0.08	0.00	**0.98 (0.94)**
FAIRFOOD 18	0.06	− 0.02	− 0.03	**0.92 (0.93)**	0.01	0.04	− 0.01	**0.89 (0.92)**
FAIRFOOD 19	− 0.03	0.11	0.09	**0.71 (0.80)**	0.00	0.01	0.05	**0.86 (0.89)**
*γ*	0.83	0.87	0.57	0.76	0.89	0.93	0.69	0.73
*ω*	0.93	0.87	0.89	0.92	0.94	0.93	0.93	0.94
AVE	0.82	0.61	0.72	0.74	0.84	0.77	0.81	0.80

*AVE* average variance extracted, *γ* second-order factor loading from CFA, *1* social, *2* economic, *3* informational, *4* environmental. Standardized factor loadings are reported. Standardized factor loadings from CFA are reported in parenthesis. All standardized factor loadings in bold are significant at *p* < 0.001. The order of items corresponds to the items’ order in Table 2. *ω* and AVE are reported for the first-order latent factors

**Table 2:** Final items for the FAIRFOOD scale (English and Italian versions)

Dimension	Item (English; *Italian*)
*It is important to me that the food I buy… (Per me è importante che il cibo che acquisto…)*	
Social	…avoids exploitation of workers (such as unethical behaviour, criminal activities, and illegal hiring) (*…eviti lo sfruttamento dei lavoratori *(*comportamenti scorretti, attività illegali, e assunzioni illecite*)
	…prevents child labour (*…impedisca il lavoro minorile*)
	…ensures worker safety and respects normal working hours (*…garantisca ai lavoratori sicurezza e rispetto dei normali orari di lavoro*)
Economic	…guarantees producers a remuneration that covers production costs (*…garantisca ai produttori una remunerazione che copra i costi di produzione*)
	…ensures farmers receive a fair income even if I have to pay a higher price (*…assicuri che i produttori ricevano un reddito equo anche se ciò significa che io sosterrò un costo maggiore*)
	…ensures farmers receive a fair price for their produce from retailers (*…assicuri che gli agricoltori ricevano un giusto prezzo per i loro prodotti dai supermercati*)
	…is governed by policies which ensure farmers receive a fair price for their produce (*…sia regolato da politiche che assicurino che gli agricoltori ricevano un giusto prezzo per la loro produzione*)
Informational	…provides consumers with information about the distribution of prices between actors in the supply chain (*…fornisca ai consumatori informazioni riguardanti la distribuzione dei prezzi tra gli attori della filiera*)
	…specifies on the label the nature of the relationship between food processors/retailers with farmers (*…specifichi sull’etichetta la natura dei rapporti tra le imprese di trasformazione/distribuzione e i produttori*)
	…indicates price distribution information on labels (*…indichi sull’etichetta informazioni riguardo la distribuzione dei prezzi*)
Environmental	…uses sustainable packaging (*…usi imballaggi sostenibili*)
	…ensures proper and responsible water management (*…assicuri una corretta e responsabile gestione dell’acqua*)
	…ensures proper and responsible waste management (*…assicuri una corretta e responsabile gestione dei rifiuti*)
	…preserves biodiversity (*…preservi la biodiversità*)

#### Confirmatory Factor Analysis

We subjected the remaining 14 items to a CFA, specifying the four hypothesized dimensions as first-order factors of the second-order FAIRFOOD factor. Across studies, we used MLR estimation for the CFA. The model had a good fit to the data in the Italy (*χ*^2^ (73) = 120.24, CFI = 0.98, TLI = 0.98, RMSEA = 0.04, SRMR = 0.04) and the UK (*χ*^2^ (73) = 127.38, CFI = 0.98, TLI = 0.97, RMSEA = 0.05, SRMR = 0.04) samples. Supporting convergent validity, all items loaded significantly and substantially on their respective dimensions (*λ* > 0.70, *p* < 0.001). As detailed in Table 2, the coefficient omega (*ω*) estimated for each dimension exceeded 0.80, supporting the dimensions’ reliability. The Average Variance Extracted (AVE) was greater than 0.50 for each FAIRFOOD dimension (Fornell & Larcker, 1981), meaning that each first-order latent factor accounted for the majority of the variance in its indicators. The second-order loadings were also significant and substantial, ranging from 0.57 to 0.87 (*M* = 0.76) in Italy, and from 0.69 to 0.93 in the UK (*M* = 0.82). Supporting reliability, the coefficient omega for the second-order model (*ω*_L1_) was 0.81 and 0.87 in the Italian and the UK samples, respectively. The AVE for the second-order construct was above 0.50 (Italy: AVE = 0.59; UK: AVE = 0.67) indicating that a majority of the variance in the first-order dimensions is shared with the second-order latent construct (MacKenzie et al., 2011).

We contrasted our operationalization against a unidimensional model in which all 14 items were specified to load on a single latent construct. The results showed that the second-order operationalization surpassed the unidimensional one. The poor fit of the unidimensional model was evident for both the Italian (*χ*^2^ (77) = 1155.62, CFI = 0.64, TLI = 0.58, RMSEA = 0.18, SRMR = 0.12) and the UK sample (*χ*^2^ (77) = 999.14, CFI = 0.65, TLI = 0.58, RMSEA = 0.19, SRMR = 0.11). The scaled difference chi-square (Δ*χ*^2^) tests (Satorra & Bentler, 2001) validated our second-order operationalization. The tests revealed that our model fitted the data significantly better than the unidimensional model for both the Italian (Δ*χ*2 [4] = 516.38, *p* < 0.001) and UK samples (Δ*χ*^2^ [4] = 311.47, *p* < 0.001).

Further justification for our second-order reflective operationalization comes from the moderate to high correlations among the dimensions across the studies (Johnson et al., 2011; Law et al., 1998). Specifically, subjecting a four-factorial correlated model in a CFA in Study 1 resulted in the correlations among dimensions ranging from 0.45 to 0.73 (*M*_*φ*_ = 0.57) in Italy and from 0.57 to 0.83 (*M*_*φ*_ = 0.66) in the UK. In Study 2, the correlations ranged from 0.53 to 0.87 (*M*_*φ*_ = 0.73) in Italy and from 0.70 to 0.93 (*M*_*φ*_ = 0.82) in the UK. In Study 4, the correlations ranged from 0.71 to 0.94 (*M*_*φ*_ = 0.82) in Italy and from 0.54 to 0.84 (*M*_*φ*_ = 0.70) in the UK. All correlations were significant at 0.001. To summarize, across the studies the second-order model performed well, and we deemed it preferable also because it is more parsimonious than the first-order model and allows for covariation among first-order dimensions (Johnson et al., 2011).

## Study 2: Measurement Validation

The objectives of this study were to: (1) confirm the psychometric properties of the hypothesized second-order factorial model using new, independent samples, (2) confirm measurement invariance, and (3) provide evidence for construct validity. Construct validity is the extent to which a measurement assesses the construct it is deemed to measure (MacKenzie et al., 2011). We sought to establish construct validity by examining the relationship of the construct within its nomological network. In doing so we formally tested for convergent and discriminant validity of our measurement in relation to various constructs identified as related to FAIRFOOD.

To assess construct validity, we started with an overview of the conceptual overlap and distinctions between FAIRFOOD and theoretically linked constructs relating to ethically conscious consumer behavior (Sudbury-Riley & Kohlbacher, 2016). We expected that FAIRFOOD would retain its uniqueness and distinctiveness (discriminant validity) but would reflect underlying similarities with theoretically related constructs (convergent validity). In particular, building on the previous literature (Balderjahn et al., 2013), we expected our construct to be positively related to FT concern (De Pelsmacker & Janssens, 2007), purchase intention toward FT products (Shih-Tse Wang & Chen, 2019), components of justice of FTOs (Shih-Tse Wang & Chen, 2019), environmental beliefs (Kilbourne & Pickett, 2008), environmental concern (Kilbourne & Pickett, 2008), and ethical consumption behavior (Toti et al., 2021). We also expected that our focal construct would demonstrate a negative correlation with FT skepticism (De Pelsmacker & Janssens, 2007). As an additional test for discriminant validity, we included FT information quality (De Pelsmacker & Janssens, 2007) which does not share a strong theoretical link with the focal construct and hence should demonstrate weak to null correlation with it.

### Participants, Procedure, and Measurements

We recruited 334 adults in Italy using Prolific. After removing seven participants who failed the attention check, the Italian sample consisted of 327 adults (*M*_age_ = 29.94; SD_age_ = 8.88; 48.01% female; 49.24% male). In the UK, a sample of 423 participants on Prolific agreed to participate. After removing five participants who failed an attention check, the final UK sample consisted of 418 participants (*M*_age_ = 36.39, SD_age_ = 12.03; 72.73% female, 26.56% male). Participants responded to the FAIRFOOD measurement in addition to the following measurements from the existing literature derived or adopted for this study (see Web Appendix for items): FT concern, FT skepticism, FT information quality, purchase intention toward FT products, components of justice of FT organizations, environmental belief, environmental concern, and ethical consumption behavior, as well as social media self-control failure as a marker variable. Measurements were presented in a randomized order. Participants then completed a socio-demographic survey. We developed Italian versions of these measurements using the translation and back translation procedure of Brislin, (1970), as discussed previously.

### Analysis and Results

#### Confirmatory Factor Analysis of FAIRFOOD

**Fig. 1:**
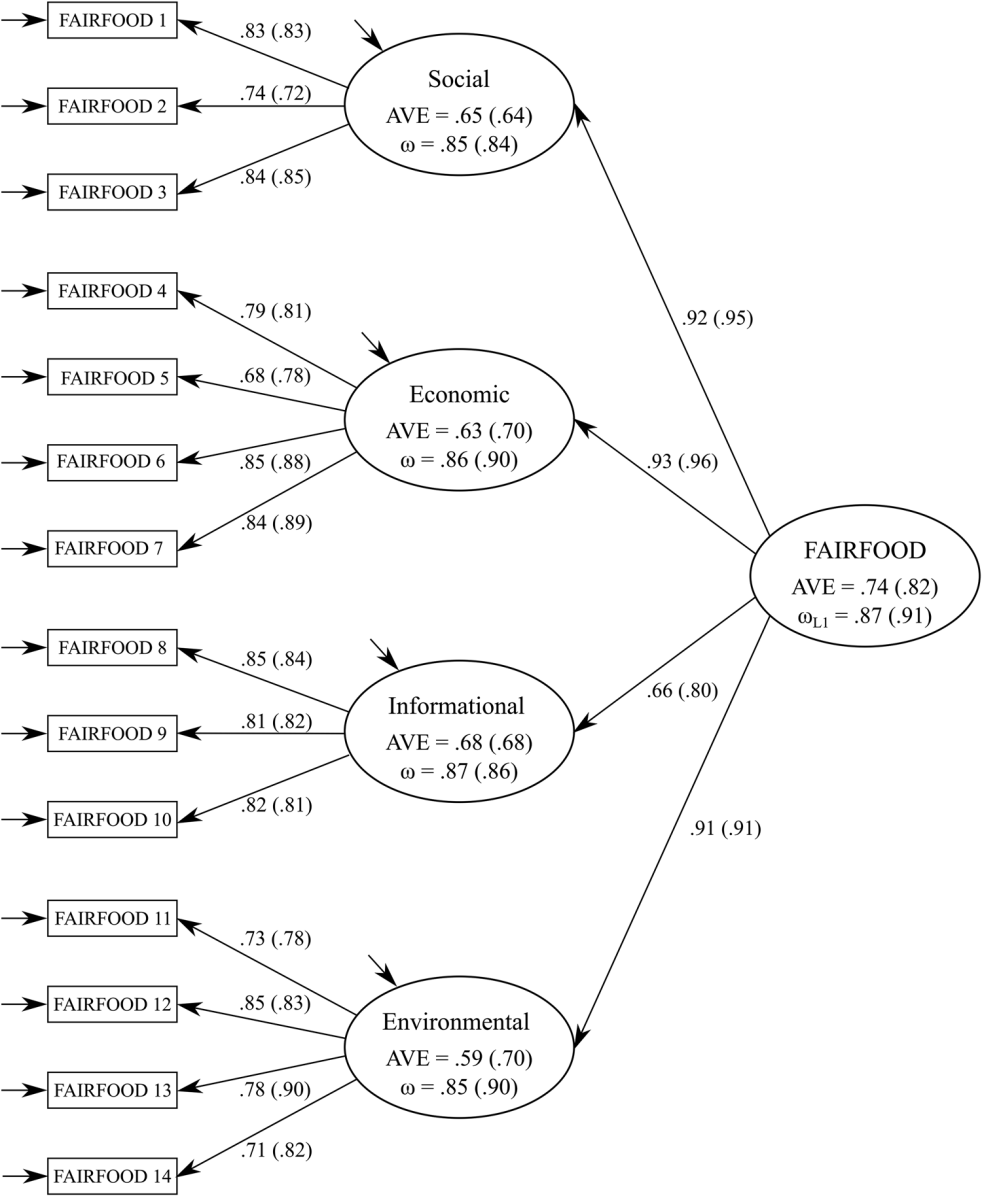
Graphical representation of the second-order model of FAIRFOOD. *Note*: FAIRFOOD = consumers’ disposition toward fairness in food supply chains. Values without parentheses correspond to the Italian sample and in parentheses to the UK sample. All first-order and second-order factor loadings are significant at *p* < 0.001

We first performed a CFA for each sample and tested a hypothesized reflective second-order structure in which we modelled FAIRFOOD as the second-order factor with four dimensions (social, economic, informational, and environmental) as first-order reflective factors (see Fig. 1). Replicating Study 1, in both samples, the second-order factorial model exhibited a good fit to the data (Italy: *χ*^2^ (73) = 76.85, CFI = 1.00, TLI = 1.00, RMSEA = 0.01, SRMR = 0.03; UK: *χ*^2^ (73) = 168.07, CFI = 0.97, TLI = 0.96, RMSEA = 0.06, SRMR = 0.04), further supporting our conceptualization that the constitutive dimensions were linked to a common second-order construct of fairness. The second-order factor loadings were statistically significant at 0.001 and substantive in size, ranging from 0.66 to 0.93 (*M* = 0.86) in the Italian sample, and from 0.80 to 0.96 in the UK sample (*M* = 0.91), indicating that the first-order factors are well explained by the second-order factor. Likewise, the individual items were well explained by their respective first-order factors, as indicated by their substantial and significant factor loadings (see Fig. 1). Supporting the measurement’s reliability, the coefficient omega for the second-order model was well above 0.80 for Italy (*ω*_L1_ = 0.87) and the UK (*ω*_L1_ = 0.91). Furthermore, the AVE for the second-order construct was 0.74 and 0.82 in the Italian and the UK samples, respectively.

Following Netemeyer et al., (2003), we tested our operationalization of the fairness construct by comparing our conceptually based second-order model with an alternative unidimensional model. The second-order operationalization outperformed the unidimensional model as demonstrated by the poor model fit for the latter in the Italian (*χ*^2^ (77) = 349.36, CFI = 0.85, TLI = 0.83, RMSEA = 0.10, SRMR = 0.08) and UK (*χ*^2^ (77) = 408.24, CFI = 0.89, TLI = 0.87, RMSEA = 0.10, SRMR = 0.06) samples. Likewise, the Δ*χ*^2^ supported our second-order operationalization by demonstrating that the model was significantly better than the unidimensional model in both samples (Italy: Δ*χ*^2^ [4] = 152.44, *p* < 0.001; UK: Δ*χ*^2^ [4] = 126.22, *p* < 0.001). We also tested the second-order four-dimensional operationalization against 10 alternative models in which two or three of the original dimensions were combined into a single factor. The results (see Web Appendix) indicate that the second-order four-dimensional model represents the data more appropriately than all alternative models.^1^

#### Measurement Invariance of FAIRFOOD

Measurement invariance concerns whether the measurement holds the same meaning for members of different groups and is a prerequisite for future comparison of groups with respect to a latent trait (Vandenberg & Lance, 2000). We performed measurement invariance tests across cultures (Italy vs. UK) and gender (women vs. men) allowing us to determine whether the same construct of disposition towards fairness in agri-food supply chains is being measured across these groups. We followed the procedure of Rudnev et al., (2018) in testing a series of restrictive hierarchical models using a multi-group CFA. In comparing nested models, we performed scaled difference chi-square tests (Satorra & Bentler, 2001). However, since Δ*χ*^2^ tests are sensitive to a large sample size, i.e., over rejection of invariance tests (Putnick & Bornstein, 2016), we also based our decision on the combination of the overall model fit, and changes in CFI, RMSEA and SRMR. We used the following criteria of model fit change: 0.01 for ΔCFI, and 0.015 for ΔRMSEA and ΔSRMR (Putnick & Bornstein, 2016).

First, we performed invariance tests at the country level. An unrestricted second-order model exhibited good fit to the data (*χ*^2^ (146) = 245.92, CFI = 0.98, TLI = 0.97, RMSEA = 0.04, SRMR = 0.03), meaning that each construct was measured by the same items in both the Italian and UK samples. Model 2 tested the invariance of first-order factor loadings and was nested within Model 1. As can be seen in Table 3, the Δ*χ*^2^ between the models was non-significant (∆*χ*^2^[10] = 14.52, *p* = 0.15) and ΔCFI, ΔRMSEA and ΔSRMR were small (< 0.01), indicating invariance of the first order factor loadings across the groups. Supporting metric invariance of second-order factor loadings (Model 3), the Δ *χ*^2^ test between Model 2 and Model 3 was not significant (∆*χ*^2^[3] = 5.67, *p* = 0.13) with marginal values for ΔCFI, ΔRMSEA and ΔSRMR (< 0.01). These findings indicate the equivalent meaning of the fairness construct across Italy and the UK, suggesting its suitability across food cultures.

**Table 3:** Results of invariance tests (Study 2)

	Models	*χ*^2^ (df)	Δ*χ*^2^(Δdf)	CFI	RMSEA	SRMR	ΔCFI	ΔRMSEA	ΔSRMR
*Italy vs. UK*									
M1: Configural invariance	–	245.92 (146)	–	0.979	0.043	0.034	–	–	–
M2: Metric invariance of the first-order factors	M2: M1	260.82 (156)	14.52 (10)^n.s.^	0.978	0.042	0.041	0.001	0.001	0.007
M3: Metric invariance of the first- and second- order factors	M3: M2	266.51 (159)	5.67 (3)^n.s.^	0.978	0.043	0.047	0.000	0.001	0.006
M4: Scalar invariance of the first-order factors	M4: M3	320.44 (169)	68.20 (10)**	0.969	0.049	0.051	0.009	0.006	0.004
M5: Scalar invariance of the first- and second- order factors	M5: M4	373.23 (172)	63.90 (3)**	0.958	0.056	0.058	0.011	0.007	0.007
*Italy: Males vs. Females*									
M1: Configural invariance	–	163.13(146)	–	0.991	0.027	0.040	–	–	–
M2: Metric invariance of the first-order factors	M2: M1	169.92 (156)	6.17 (10)^n.s.^	0.993	0.024	0.046	0.002	0.003	0.006
M3: Metric invariance of the first- and second- order factors	M3: M2	170.16 (159)	0.53 (3)^n.s.^	0.994	0.021	0.047	0.001	0.003	0.001
M4: Scalar invariance of the first-order factors	M4: M3	187.97 (169)	19.34 (10)*	0.990	0.027	0.050	0.004	0.006	0.003
M5: Scalar invariance of the first- and second- order factors	M5: M4	189.49 (172)	1.00 (3)^n.s.^	0.991	0.025	0.050	0.001	0.002	0.000
*UK: Males vs. Females*									
M1: Configural invariance	–	263.21 (146)	–	0.964	0.062	0.040	–	–	–
M2: Metric invariance of the first-order factors	M2: M1	274.62 (156)	10.15 (10)^n.s.^	0.963	0.061	0.047	0.001	0.001	0.007
M3: Metric invariance of the first- and second- order factors	M3: M2	276.00 (159)	1.84 (3)^n.s.^	0.964	0.060	0.051	0.001	0.001	0.004
M4: Scalar invariance of the first-order factors	M4: M3	293.88 (169)	18.02 (10)^n.s.^	0.961	0.060	0.052	0.003	0.000	0.001
M5: Scalar invariance of the first- and second- order factors	M5: M4	296.59 (172)	2.03 (3)^n.s.^	0.961	0.059	0.067	0.000	0.001	0.015

Scaled *χ*^2^ and Δ*χ*^2^ are reported

*df* degrees of freedom

* *p* < 0.05

**p < 0.001

^n.s.^Non-significant (*p* > 0.05)

Model 4, nested within Model 3, tested for scalar invariance of the first-order factors. The Δ*χ*^2^ between these models was significant (∆*χ*^2^[10] = 68.20, *p* < 0.001), but ΔCFI, ΔRMSEA and ΔSRMR were below < 0.01, supporting scalar invariance of the first-order factors (see Table 3). Finally, Model 5 tested for scalar invariance of the second-order factor and was nested in Model 4. Although, the chi-square difference test between these models was significant (∆*χ*^2^[3] = 63.90, *p* < 0.001), ΔRMSEA, ΔSRMR and ΔCFI were ≤ 0.01, together suggesting no substantial deviation in model fit. These results indicate scalar invariance of the second-order factors. Taken together, the results of Models 4 and 5 imply that the means of the four first-order factors and the mean of the second-order factor of FAIRFOOD could be compared with a degree of confidence.

We then tested for measurement invariance across gender (see Table 3). In the Italian sample, we found non-significant Δ*χ*^2^ between Model 1 and Model 2 (∆*χ*^2^[10] = 6.17, *p* = 0.80), Model 2 and Model 3 (∆*χ*^2^[3] = 0.53, *p* = 0.91) with △CFI, △RMSEA and ΔSRMR between these models were ≤ 0.01. The Δ*χ*^2^ between Model 3 and Model 4 was significant (∆*χ*^2^[10] = 19.34, *p* = 0.04), but △CFI, △RMSEA and ΔSRMR were ≤ 0.01, supporting scalar invariance of the first-order factors. The Δ*χ*^2^ between Model 4 and Model 5 was non-significant (∆*χ*^2^[3] = 1.00, *p* = 0.80). As shown in Table 3, in the UK sample, the Δ*χ*^2^ across all model comparisons was non-significant and all △CFI, △RMSEA and ΔSRMR were ≤ 0.01. Taken together, the results in both the Italian and UK samples, support configural, metric and scalar invariance of FAIRFOOD across gender.

#### Measurement Model Assessment and Common Method *Bias*

Prior to examining the relationship between our measurement and other conceptually related constructs, we first assessed the measurement model in which we modelled all first-order constructs as reflective and second-order constructs as reflective-reflective. The overall measurement model had an acceptable fit to the data for Italy (*χ*^2^ (1466) = 2157.01, CFI = 0.93, TLI = 0.93, RMSEA = 0.04, SRMR = 0.06) and the UK (*χ*^2^ (1466) = 2451.02, CFI = 0.93, TLI = 0.93, RMSEA = 0.04, SRMR = 0.05). As shown in Table 4, almost all first-order indicators loaded significantly and substantially (> 0.70, *p* < 0.001) on their respective constructs, confirming individual indicator reliability. Although a few items had loadings below 0.70, we retained them in the analysis. Supporting the measurement’s reliability, the values of *ω* were greater than 0.70 for each construct (Nunnally & Bernstein, 1994). For all constructs, AVE exhibited acceptable values above the required threshold of 0.50, indicating that the constructs explained more than the half of the variance of their indicators. We report the assessment of discriminant validity among the constructs below. Finally, we assessed common method bias using a CFA marker variable approach (Williams et al., 2010), using social media self-control failure as the marker variable, as on conceptual and theoretical grounds it should not be strongly correlated with FAIRFOOD. The results (see Web Appendix) demonstrate that for both the Italian and the UK samples, no significant difference existed between Method-U and Method-R, suggesting that common method variance did not exert any significant influence on the relationship between variables. The results, hence, confirm that common method bias is not a serious concern in the study.

**Table 4:** Psychometric properties of the measurements model (Study 2)

Latent construct	Source	Item code	Italy			UK		
			*λ*	*ω*	AVE	*λ*	*ω*	AVE
*First-order constructs*								
FT concern	De Pelsmacker & Janssens, (2007)	CNR 1	0.88	0.73	0.54	0.78	0.73	0.50
		CNR 2	0.81			0.69		
		CNR 3	0.44			0.64		
FT scepticism	De Pelsmacker & Janssens, (2007)	SCP 1	0.79	0.80	0.57	0.76	0.79	0.57
		SCP 2	0.70			0.86		
		SCP 3	0.78			0.61		
Information quality	De Pelsmacker & Janssens, (2007)	INQ 1	0.76	0.75	0.52	0.70	0.80	0.57
		INQ 2	0.62			0.77		
		INQ 3	0.77			0.79		
Purchase intention toward FT products	Shih-Tse Wang & Chen, (2019)	PI 1	0.86	0.94	0.83	0.90	0.92	0.80
		PI 2	0.95			0.88		
		PI 3	0.91			0.91		
Perceived distributive justice of FTOs	Shih-Tse Wang & Chen,(2019)	DSJ 1	0.92	0.92	0.79	0.90	0.90	0.76
		DSJ 2	0.92			0.94		
		DSJ 3	0.83			0.77		
Perceived procedural justice of FTOs	Shih-Tse Wang and Chen (2019)	PSJ 1	0.83	0.88	0.70	0.87	0.91	0.77
		PSJ 2	0.82			0.87		
		PSJ 3	0.87			0.88		
Perceived interactional justice of FTOs	Shih-Tse Wang & Chen, (2019)	ISJ 1	0.91	0.88	0.71	0.92	0.87	0.70
		ISJ 2	0.88			0.90		
		ISJ 3	0.73			0.68		
Environmental belief	Kilbourne & Pickett, (2008)	EBV 1	0.85	0.79	0.52	0.89	0.83	0.56
		EBV 2	0.68			0.73		
		EBV 3	0.56			0.68		
		EBV 4	0.75			0.67		
Environmental concern	Kilbourne & Pickett, (2008)	ECR 1	0.81	0.86	0.61	0.84	0.89	0.68
		ECR 2	0.77			0.83		
		ECR 3	0.81			0.81		
		ECR 4	0.74			0.82		
Marker	Du et al., (2018)	MRK 1	0.86	0.86	0.66	0.90	0.93	0.81
		MRK 2	0.76			0.89		
		MRK 3	0.82			0.92		
*Second-order constructs*								
FAIRFOOD		SOC	0.91	0.87	0.74	0.95	0.91	0.83
		ECO	0.91			0.95		
		INF	0.67			0.81		
		ENV	0.94			0.93		
Ethical consumption behaviour	Toti et al., (2021)	POL	0.85	0.80	0.72	0.92	0.84	0.76
		SOC	0.83			0.79		
		ENV	0.86			0.90		

*λ* standardized loadings. For second-order constructs, second-order standardized loadings are reported. *FAIRFOOD* consumers’ disposition toward fairness in food supply chains, *SOC* social, *ECO* economical, *INF* informational, *POL* political. All loadings are significant at *p* < 0.001 level

#### Assessment of Construct Validity

The correlations in Tables 5 and 6 reveal the extent to which the predictions regarding convergent and discriminant validity were supported for FAIRFOOD. Regarding convergent validity, we found that FAIRFOOD was strongly and significantly related to FT concern, FT purchase intentions, ethical consumption behavior, environmental beliefs, environmental concern, and three dimensions of justice of FTOs (distributive, procedural and interactional) with correlations ranging from 0.38 to 0.75 (all *p* < 0.001) in the Italian sample, and from 0.27 to 0.77 (all *p* < 0.001) in the UK sample. All these relationships were in the expected positive direction. As predicted, we found a negative and significant relationship between FAIRFOOD and FT skepticism (Italy: *φ* = − 0.33, *p* < 0.001; UK:* φ* =  − 0.27, *p* < 0.001).

**Table 5:** Descriptive statistics, correlations, and HTMT2 ratios for the Italian sample (Study 2)

Latent construct	1	2	3	4	5	6	7	8	9	10	11	12
1. FAIRFOOD	**(0.86)**	0.60	0.31	0.19	0.49	0.39	0.44	0.51	0.44	0.54	0.77	0.05
2. FT concern	0.57**	**(0.73)**	0.40	0.13	0.67	0.59	0.67	0.65	0.40	0.50	0.69	0.04
3. FT scepticism	− 0.33**	− 0.59**	**(0.75)**	0.52	0.56	0.47	0.57	0.50	0.33	0.36	0.40	0.03
4. FT information quality	− 0.23**	− 0.35**	0.56**	**(0.72)**	0.18	0.38	0.33	0.36	0.11	0.12	0.19	0.18
5. FT purchase intentions	0.51**	0.68**	− 0.56**	− 0.26**	**(0.91)**	0.55	0.61	0.58	0.42	0.46	0.60	0.03
6. Distributive justice of FTOs	0.38**	0.63**	− 0.46**	− 0.39**	0.55**	**(0.89)**	0.85	0.86	0.27	0.28	0.42	0.06
7. Procedural justice of FTOs	0.42**	0.71**	− 0.56**	− 0.36**	0.60**	0.84**	**(0.84)**	0.90	0.34	0.43	0.52	0.04
8. Interactional justice of FTOs	0.47**	0.64**	− 0.49**	− 0.36**	0.57**	0.84**	0.88**	**(0.84)**	0.39	0.43	0.56	0.03
9. Environmental belief	0.45**	0.40**	− 0.34**	− 0.13^n.s.^	0.44**	0.27**	0.34**	0.39**	**(0.72)**	0.82	0.49	0.21
10. Environmental concern	0.56**	0.51**	− 0.37**	− 0.21**	0.45**	0.29**	0.44**	0.42**	0.80**	**(0.78)**	0.62	0.15
11. Ethical consumption behaviour	0.75**	0.65**	− 0.41**	− 0.28**	0.60**	0.42**	0.52**	0.52**	0.48**	0.63**	**(0.85)**	0.05
12. Social media self-control failure^a^	0.05^n.s.^	0.03^n.s.^	0.00^n.s.^	0.19**	0.05^n.s.^	0.01^n.s.^	0.02^n.s.^	0.05^n.s.^	0.21**	0.20**	0.07^n.s.^	**(0.81)**
*M*	5.88	5.02	3.03	3.97	5.69	5.10	5.18	5.13	6.37	6.30	5.07	3.25
SD	0.75	0.96	1.17	1.11	1.03	0.94	0.95	0.95	0.69	0.79	0.91	0.97

The square root of AVE of each construct is shown in bold and on the diagonal in parentheses. HTMT2 ratios are reported above the diagonal

*FAIRFOOD* consumers’ disposition toward fairness in food supply chains

**p < 0.001

^n.s..^Non-significant (*p* > 0.05)

^a^Marker variable

**Table 6:** Descriptive statistics, correlations, and HTMT2 ratios for the UK sample (Study 2)

Latent construct	1	2	3	4	5	6	7	8	9	10	11	12
1. FAIRFOOD	**(0.91)**	0.62	0.20	0.02	0.52	0.30	0.26	0.39	0.44	0.56	0.79	0.01
2. FT concern	0.63**	**(0.71)**	0.14	0.12	0.69	0.26	0.30	0.35	0.51	0.61	0.62	0.25
3. FT scepticism	− 0.27**	− 0.26**	**(0.75)**	0.64	0.43	0.50	0.47	0.51	0.07	0.20	0.21	0.14
4. FT information quality	− 0.02^n.s.^	0.00^n.s.^	0.64**	**(0.75)**	0.15	0.32	0.36	0.30	0.10	0.04	0.05	0.03
5. FT purchase intentions	0.52**	0.70**	− 0.46**	− 0.15*	**(0.89)**	0.50	0.50	0.55	0.40	0.48	0.68	0.17
6. Distributive justice of FTOs	0.31**	0.36**	− 0.53**	− 0.31**	0.51**	**(0.87)**	0.74	0.80	0.09	0.20	0.36	0.03
7. Procedural justice of FTOs	0.27**	0.37**	− 0.50**	− 0.36**	0.50**	0.75**	**(0.88)**	0.86	0.12	0.17	0.31	0.04
8. Interactional justice of FTOs	0.38**	0.43**	− 0.54**	− 0.31**	0.53**	0.79**	0.83**	**(0.84)**	0.15	0.25	0.39	0.03
9. Environmental belief	0.48**	0.54**	− 0.10	0.09^n.s.^	0.44**	0.13*	0.15*	0.17*	**(0.75)**	0.84	0.42	0.18
10. Environmental concern	0.57**	0.60**	− 0.22**	0.03^n.s.^	0.48**	0.21**	0.18*	0.26**	0.87**	**(0.82)**	0.63	0.18
11. Ethical consumption behaviour	0.77**	0.61**	− 0.30**	− 0.06 ^n.s.^	0.69**	0.36**	0.32**	0.38**	0.46**	0.63**	**(0.87)**	0.03
12. Social media self-control failure^a^	0.02^n.s.^	0.24**	0.15*	0.03^n.s.^	0.17*	0.00^n.s.^	0.02^n.s.^	0.03^n.s.^	0.20**	0.18*	0.05^n.s.^	**(0.90)**
*M*	5.41	5.32	3.46	4.31	5.60	5.04	5.06	5.08	6.14	5.86	4.71	3.12
SD	0.98	0.93	1.22	1.14	1.08	1.03	0.94	0.91	0.77	1.00	0.99	1.13

The square root of AVE of each construct is shown in bold and on the diagonal in parentheses. HTMT2 ratios are reported above the diagonal

*FAIRFOOD* consumers’ disposition toward fairness in food supply chains

^a^Marker variable

*p < 0.05

**p < 0.001

^n.s..^Non-significant (*p* > 0.05)

We used a combination of the criterion of Fornell & Larcker, (1981) and HTMT2 (Roemer et al., 2021) to assess discriminant validity. Results are reported in Tables 5 and 6. Following the Fornell–Larcker criterion, the square root of AVE for FAIRFOOD was greater than the correlation between the respective constructs, supporting discriminant validity of the construct. Next, we assessed discriminant validity with the HTMT2 method using both a conservative critical value of 0.85 and a more liberal value of 0.90 (Henseler et al., 2015). Note, to calculate HTMT2 ratios, we estimated a new measurement model in which we modelled two second-order constructs (FAIRFOOD and ethical consumption behavior) based on the two-stage approach (e.g., Sarstedt et al., 2019). Providing additional evidence of discriminant validity, the HTMT2 ratios between FAIRFOOD and other studied constructs ranged from 0.19 to 0.77 in the Italian sample and from 0.02 to 0.79 in the UK sample, well below the threshold of 0.85 (Henseler et al., 2015). Likewise, Fornell–Larcker and HTMT2 criteria supported discriminant validity for other constructs.^2^ Finally, we examined the relationship between FAIRFOOD and FT information quality, which revealed a small and negative correlation (*φ* =  − 0.23, *p* < 0.001) in the Italian sample, and a non-significant correlation in the UK sample (*φ* =  − 0.02, *p* > 0.05). Taken together, the results of this study provide evidence of the construct validity of FAIRFOOD.

## Study 3: Strengthening Construct Validity

In this study, we sought to strengthen the construct validity of FAIRFOOD by further examining its relationships within its nomological network. While Study 2 established an empirical connection between FAIRFOOD and constructs related to ethically conscious consumer behavior, the present study primarily aimed to investigate the theoretical links between FAIRFOOD and selected psychological (personality and moral identity) constructs.

We expected that moral identity would positively relate to a disposition towards fairness in agri-food supply chains. Individuals with a strong moral identity place a higher priority on ensuring their actions reflect their moral values compared to those with a weaker moral identity (Aquino & Reed, 2002; Reed & Aquino, 2003). This alignment with ethical identity is likely to extend to their views on fairness in the agri-food supply chain, as they are more likely to prioritize fair treatment of all actors involved due to their moral commitments being central to their self-definition. Likewise, we expected FAIRFOOD to positively relate with ethical sensitivity which refers to “a predisposition of an individual to consider one or more ethical aspects in decision-making” (Toti & Moulins, 2017, p. 7). Ethical sensitivity significantly affects ethical judgments and consumer behavior (Toti et al., 2021), and hence, we anticipated that individuals with elevated ethical sensitivity place greater importance on fairness within agri-food supply chains.

FAIRFOOD is expected to show a positive correlation with self-efficacy in ethical consumption. Following previous studies on task-specific and domain-specific self-efficacy beliefs (Grether et al., 2018), self-efficacy in ethical consumption refers to an individual’s belief in their ability to engage effectively in ethical consumption behaviors. This belief is likely to foster a proactive approach towards seeking fairer outcomes and advocating for ethical practices. We argue that consumers who possess a high level of self-efficacy in this area are more inclined to value fairness in agri-food supply chains. Drawing on Cacioppo et al., (1984), we also anticipate a positive correlation between the Need for Cognition (NFC)—an individual's enjoyment of effortful cognitive tasks—and FAIRFOOD. This connection is supported by evidence that individuals with a high NFC are likely to engage in moral behaviors (Strobel et al., 2017) and weigh ethical decisions based on the implications and societal norms surrounding an issue (e.g., Singer et al., 1998).

We expected a positive convergence between FAIRFOOD and two personality traits—agreeableness and conscientiousness. Agreeableness, characterized by qualities such as kindness, gentleness, trust, honesty, altruism, and warmth (McCrae & Costa, 1987) is indicative of individuals who are caring and empathetic towards others (Kalshoven et al., 2011). Given these traits, we posit that agreeable individuals are likely to exhibit a heightened disposition for the fair treatment of all actors within the supply chain. Conscientious individuals, known for their careful deliberation before acting and strong adherence to moral obligations and responsibilities (Costa & McCrae, 1992), embody traits of responsibility, dependability, and deliberation (Kalshoven et al., 2011). This duty-focused aspect of conscientiousness suggests they are predisposed to ethical behavior, benefiting not just themselves but others as well (Moon, 2001).

Finally, we also expanded our construct's nomological network by examining its relationship with two outcomes unrelated to FT certification: boycott and negative word-of-mouth intentions. Consistent with previous studies on consumer activism and reactions to moral wrongdoings (e.g., Khamitov et al., 2020; Klein, 1991), we expected that individuals with a stronger disposition towards fairness in agri-food supply chains are more likely to engage in boycotts and negative WOM as tools to express their ethical stances and to advocate for fair treatment across the supply chain.

### Participants, Procedure, and Measurements

We determined the sample size suitable for correlational analysis prior to data collection using power analysis. We aimed to collect responses from at least 255 participants that would provide 90% power (*α* = 0.05; two-tailed) to detect an effect size of *r* =  ± 0.20. In both Italy and the UK, we recruited 300 participants via Prolific. We excluded 13 Italian participants and ten UK participants who failed an attention check or had missing values in responses. Thus, the final samples consisted of 287 participants in Italy (*M*_age_ = 33.29; SD_age_ = 10.52; 41.46% female; 55.40% male) and 290 participants in the UK (*M*_age_ = 41.57; SD_age_ = 13.51; 66.21% female; 32.76% male). A sensitivity power analysis indicated that both final samples provide 90% power to detect a small effect size (*r* =  ± 0.19) with *α* = 0.05 (two-tailed).

In total we measured, ten constructs. These included: FAIRFOOD, moral identity (Aquino & Reed, 2002), self-efficacy in ethical consumption; ethical sensitivity (Toti et al., 2021); need for cognition (Lins De Holanda Coelho et al., 2020); the personality traits of agreeableness and conscientiousness (Maples-Keller et al., 2019) and social desirability (Protzko et al., 2019). Participants were also presented with a scenario task designed to assess their boycott and negative WOM intentions. This task required them to envision a situation where a favoured food brand is implicated in unfair treatment towards supply chain actors such as farmers, suppliers, and consumers. Following this task, they indicated their boycott (Zarantonello et al., 2016) and negative WOM intentions (Zarantonello et al., 2016). We used the same measure as a marker variable as in the previous study. The order of all measures was randomized. The study concluded with participants completing a socio-demographic survey. The list of items can be found in the Web Appendix. The measures demonstrated good reliability (see Tables 7, 8) and were aggregated for subsequent analysis.

**Table 7:** Descriptive statistics, reliability, HTMT ratios and partial correlations for the Italian sample (Study 3)

	1	2	3	4	5	6	7	8	9
1. FAIRFOOD		0.42	0.20	0.40	0.54	0.32	0.45	0.51	0.51
2. Moral identity	0.36***		0.26	0.31	0.61	0.50	0.69	0.26	0.22
3. Need for cognition	0.19***	0.19***		0.29	0.33	0.38	0.22	0.09	0.16
4. Self-efficacy (EC)	0.38***	0.26***	0.25***		0.35	0.28	0.28	0.34	0.36
5. Ethical sensitivity	0.50***	0.48***	0.26***	0.37***		0.32	0.72	0.40	0.38
6. Conscientiousness	0.28***	0.33***	0.34***	0.27***	0.18**		0.45	0.12	0.16
7. Agreeableness	0.36***	0.42***	0.03	0.12*	0.59***	0.07		0.23	0.19
8. Boycott intention	0.47***	0.24***	0.11	0.32***	0.39***	0.09	0.28***		0.79
9. Negative WOM	0.47***	0.25***	0.17**	0.35***	0.39***	0.20***	0.19***	0.69***	
*ω*	0.84	0.77	0.86	0.84	0.75	0.82	0.77	0.91	0.89
*M*	5.93	4.23	3.59	4.38	5.45	3.66	3.78	5.29	5.24
SD	0.78	0.56	0.80	1.07	0.75	0.62	0.49	1.32	1.10

Partial correlations controlling for social desirability (*ω* = 0.70; *M* = 3.95, SD = 0.80). HTMT2 ratios are reported above the diagonal. *ω* for higher-order constructs is reported for FAIRFOOD and ethical sensitivity

*FAIRFOOD* consumers’ disposition toward fairness in food supply chains, *EC* ethical consumption

*p < 0.05

**p < 0.01

***p < 0.001

**Table 8:** Descriptive statistics, reliability, HTMT ratios and partial correlations for the UK sample (Study 3)

	1	2	3	4	5	6	7	8	9
1. FAIRFOOD		0.45	0.19	0.63	0.53	0.19	0.31	0.60	0.60
2. Moral identity	0.37***		0.14	0.21	0.70	0.32	0.61	0.34	0.22
3. Need for cognition	0.16**	0.08		0.28	0.22	0.55	0.20	0.17	0.12
4. Self-efficacy (EC)	0.56***	0.14*	0.23***		0.44	0.21	0.23	0.53	0.49
5. Ethical sensitivity	0.49***	0.55***	0.18**	0.38***		0.25	0.67	0.39	0.40
6. Conscientiousness	0.10	0.15**	0.41***	0.09	0.02		0.31	0.10	0.09
7. Agreeableness	0.30***	0.40***	0.05	0.18**	0.55***	0.01		0.19	0.20
8. Boycott intention	0.57***	0.29***	0.15*	0.48***	0.38***	0.06	0.25***		0.85
9. Negative WOM	0.58***	0.25***	0.12*	0.46***	0.41***	0.00	0.22***	0.79***	
*ω*	0.90	0.76	0.85	0.87	0.77	0.81	0.77	0.93	0.92
*M*	5.31	4.40	3.37	4.30	5.44	3.82	3.97	5.10	4.73
SD	1.03	0.54	0.79	1.10	0.73	0.55	0.49	1.42	1.29

Partial correlations controlling for social desirability (*ω* = 0.70; *M* = 4.01, SD = 0.82). HTMT2 ratios are reported above the diagonal. *ω* for higher-order constructs is reported for FAIRFOOD and ethical sensitivity

*FAIRFOOD* consumers’ disposition toward fairness in food supply chains, *EC* ethical consumption

*p < 0.05

**p < 0.01

***p < 0.001

### Analysis and Results

#### Confirmatory Factor Analysis of FAIRFOOD

Replicating previous studies, in both samples, the second-order factorial model exhibited a good fit to the data (Italy: *χ*^2^ (73) = 137.69, CFI = 0.96, TLI = 0.94, RMSEA = 0.06, SRMR = 0.06; UK: *χ*^2^ (73) = 85.42, CFI = 0.99, TLI = 0.99, RMSEA = 0.02, SRMR = 0.03). The second order factor loadings were substantial and significant, ranging from 0.71 to 0.91 in the Italian and from 0.76 to 0.93 in the UK samples (all *p* < 0.001). The measurement demonstrated good reliability (Italy: *ω*_L1_ = 0.84; UK: *ω*_L1_ = 0.90) and the AVE for the second order construct exceeded 0.50 (Italy: AVE = 0.66; UK: AVE = 0.77). In addition, the results provided evidence of measurement invariance across different cultures and genders (see Web Appendix).

#### Construct Validity

Prior to the main analysis, we examined the presence of common method variance using Lindell & Whitne’s, (2001) partial correlation method. We applied the minimal observed correlation with the marker variable to adjust for the other correlations. The analysis showed that all relationships maintained their significance even after adjusting for partial correlation. This indicates that common method variance is unlikely to significantly threaten the validity of the study’s results. The bivariate and marker variable adjusted correlations are reported in the Web Appendix.

Next, we examined the partial correlation between FAIRFOOD and other studied variables, partialing out self-ratings on social desirability. The partial correlations are reported in Tables 7 and 8 for the Italian and UK samples, respectively. Supporting construct validity of our measurement, FAIRFOOD was positively and significantly related to moral identity, self-efficacy in ethical consumption, need for cognition and ethical sensitivity with correlations ranging from 0.19 to 0.50 (all *p* < 0.001) in the Italian sample, and from 0.16 to 0.56 (all *p* < 0.01) in the UK sample.

In terms of personality traits, the analyses revealed that in the Italian sample, FAIRFOOD was positively and significantly correlated with both conscientiousness (*r* = 0.28, *p* < 0.001) and agreeableness (*r* = 0.36, *p* < 0.001). However, in the UK sample, only the association with agreeableness reached significance (*r* = 0.30, *p* < 0.001). Finally, in both samples, FAIRFOOD was positively and significantly related to boycott intentions (Italy: *r *= 0.47,* p* < 0.001; UK: *r* = 0.57, *p* < 0.001) and negative WOM (Italy: *r* = 0.47, *p* <0.001; UK: *r* = 0.58, *p* < 0.001) towards a brand which engaged in unethical supply chain practices. All these correlations were in the expected positive direction. The HTMT2 ratios between studied constructs were well below 0.85, thus providing additional evidence for the discriminant validity of our scale.

We conducted multiple regression analysis to examine the relationship FAIRFOOD has with the theoretically derived antecedents (moral identity, self-efficacy, ethical sensitivity, and personality traits). As a conservative test of our model, we controlled for social desirability. In the Italian sample, the model explained substantial variance in FAIRFOOD (adjusted *R*^2^ = 0.33). Self-efficacy (*β* = 0.20, *p* < 0.01), ethical sensitivity (*β* = 0.31, *p* < 0.01), conscientiousness (*β* = 0.15, *p* = 0.01) and agreeableness (*β* = 0.14, *p* = 0.042) were all significant predictors of FAIRFOOD. Social desirability also emerged as a significant predictor (*β* =  − 0.15, *p* = 0.011). The correlation between social desirability and FAIRFOOD in the Italian sample may suggest a potential demand effect. However, this correlation might also reflect a deeper, culturally ingrained value placed on fairness and ethical practices in Italy, reflecting that Italian participants regard fairness in food supply chains as a societal priority. This is further supported by Italy's strong tradition of local and sustainable food production, such as the Slow Food movement, which emphasizes ethical sourcing and community engagement. Additionally, Italy has a robust network of box schemes, short supply chains, and farmers' markets, all of which prioritize direct relationships between producers and consumers, ensuring transparency and fairness. Therefore, while social desirability might influence responses, it is plausible that the correlation reflects an authentic commitment to fair food practices prevalent in Italian culture. The relationship between moral identity and FAIRFOOD was non-significant (*β* = 0.07, *p* > 0.10).

The results reveal that for the UK sample, moral identity (*β* = 0.17, *p* = 0.003), self-efficacy (*β* = 0.44, *p* < 0.001), ethical sensitivity (*β* = 0.21, *p* = 0.001) predict variance in FAIRFOOD (adjusted *R*^2^ = 0.43), whereas agreeableness (*β* = 0.05, *p* > 0.10) and conscientiousness (*β* = 0.04, *p* > 0.10) do not. Social desirability has a non-significant effect on FAIRFOOD (*β* =  − 0.03, *p* > 0.10) in the UK case.

## Study 4: Assessing Predictive and Nomological Validity

The objectives of this study were to (1) confirm the psychometric properties of the hypothesized second-order factorial model using new independent samples, and (2) provide further evidence for construct validity by assessing the predictive and nomological validity of the FAIRFOOD scale.

### Direct Relationships

Trait theory, sometimes referred to as disposition theory, argues that differences in aspects of personality (traits) help explain variations in behavior (Baumeister et al., 2006), including variations in pro-social behavior (Hilbig et al., 2014). This assumes dispositions are relatively stable over time, in contrast to moods, and differ across individuals, affecting their behavior in varied situations (Costa & McCrae, 1998). Individuals differ in terms of their ethical traits (Verwey & Asare, 2022), determining engagement in (un)ethical behaviors (Kidder, 2005). While not directly measuring consumers’ dispositions toward fairness, extant research on ethical purchasing measures the impacts of general ethical attitudes or attitudes toward specific forms of ethical consumption on purchasing behavior (Balabanis & Diamantopoulos, 2016; De Pelsmacker & Janssens, 2007; Zerbini et al., 2019) and commitment (Eisingerich & Rubera, 2010), where commitment refers to a perceived feelings of attachment to an object or situation (Germann et al., 2014).

Based on trait theory, we expect that those consumers with a stronger disposition toward fairness in agri-food supply chains, will be more willing to purchase goods which seek to embody that disposition (e.g., FT certified products). Secondly, dispositions affect commitments (Kemp et al., 2014), so that consumers become psychologically attached to brands and products that are consistent with their dispositions (such as toward fairness) and repulsed by inconsistent or incompatible objects (Nijssen et al., 2003). Consequently, a stronger disposition toward fairness in agri-food supply chains should positively affect commitment towards FT certified products. Finally, a positive individual disposition towards fairness in agri-food supply chains will also affect consumer engagement (Giampietri et al., 2016), so that:

**H1**. Disposition towards fairness in agri-food supply chains positively influences willingness to purchase FT certified products.

**H2**. Disposition towards fairness in agri-food supply chains positively influences commitment to FT certified products.

**H3**. Disposition towards fairness in agri-food supply chains positively influences frequency of engagement with FT products and activities.

Dispositions differ from emotions as the former are aspects of personality, while emotions are transient mental states. Psychological research identifies, at a high level of abstraction, a two-dimensional model of emotions, namely positive and negative affect (De Raad & Kokkonen, 2000). The nature of a person’s dispositions affects their emotional responses to a particular situation or behavior, with engagement in behavior consistent with that trait generating a positive emotional response (Snippe et al., 2018). Conversely, engagement in behavior inconsistent with a strongly held trait is likely to generate negative emotions. Based on an adaptation of the positive and negative affect schedule (PANAS) scale formulated by Watson et al., (1988), we thus test the hypothesis that a person with a positive disposition towards fairness in agri-food supply chains experiences positive emotions when purchasing products which seek to embody that disposition. (e.g., FT certified goods). Likewise, regarding negative emotions, we predict that a person with a positive disposition toward fairness in agri-food supply chains is likely to experience negative emotions when not purchasing products consistent with that disposition. Consequently, we test:

**H4**. Disposition towards fairness in agri-food supply chains positively influences the experience of positive emotions when buying FT products.

**H5**. Disposition towards fairness in agri-food supply chains positively influences the experience of negative emotions when not buying FT products.

### Indirect Relationship (Mediators)

In relationship marketing theory, commitment and emotions are key mediating constructs (Garbarino & Johnson, 1999; Morgan & Hunt, 1994; Taylor et al., 2016), which we discuss in turn. Affective commitment, as discussed above, refers to perceived feelings of attachment to an object or situation (Germann et al., 2014). Dispositions affect commitments, in that consumers become attached to objects that are consistent with their dispositions. A commitment generates a desire to maintain a relationship that is psychologically rewarding for the consumer (Morgan & Hunt, 1994), strengthening the customer’s identification with the product or firm (Evanschitzky et al., 2006). In turn, affective commitments, ‘characterized by a desire-based attachment’ (Evanschitzky et al., 2006, p. 1209), have a positive effect on behavioral outcomes such as purchase intentions, loyalty, and engagement (Barari et al., 2021; Evanschitzky et al., 2006; Gustafsson et al., 2005). Applying this reasoning to fairness in agri-food supply chains suggests that:

**H6**. Commitment to FT certified products mediates the relationship between consumer disposition towards fairness in agri-food supply chains and willingness to purchase FT products.

**H7**. Commitment to FT certified products mediates the relationship between consumer disposition towards fairness in agri-food supply chains and frequency of engagement with FT products.

The generation of affective commitments will be strengthened by positive emotions, and trait theory acknowledges that emotions can mediate relations between traits and commitments (Panaccio & Vandenberghe, 2012). Specifically, dispositions create mental states (positive or negative affects) which in turn influence attachments to objects (Kokkonen & Pulkkinen, 2001). Consequently, moral dispositions generate discrete emotions, which can be either positive or negative, which shape moral judgments and commitments (Agnihotri et al., 2012). Panaccio & Vandenberghe, (2012) demonstrate the existence of such a mediating relationship in a workplace context, whereby negative and positive emotions, as measured according to the scale of Watson et al., (1988), mediate the relationship between neuroticism and organizational commitment. Applying this to the context of fairness within agri-food supply chains suggests:

**H8**. Positive emotions mediate the relationship between consumer disposition towards fairness in agri-food supply chains and commitment to FT products.

**H9**. Negative emotions mediate the relationship between consumer disposition towards fairness in agri-food supply chains and commitment to FT products.

### Participants, Procedure, and Measurements

Participants were 349 adults in Italy (*M*_age_ = 30.30; SD_age_ = 9.02; 48.71% female; 49.86% male) and 350 adults in the UK (*M*_age_ = 46.73; SD_age_ = 15.55; 51.71% female; 48% male) recruited using Prolific. Participants responded to the 14-item FAIRFOOD measurement in addition to measurements of commitment towards FT certified products, experience of negative emotions when not buying FT certified products, experience of positive emotions when buying FT certified products, willingness to purchase FT certified products, and frequency of engagement with certified FT products presented in a randomized order. Next, participants then completed a demographic survey. Like Study 2, we derived and adapted all measurements from the existing literature, and we used the same translation procedure to develop the Italian version (see Web Appendix for items).

### Analysis and Results

#### Statistical Modeling Technique

We used variance based Partial Least Squares Structural Equation Modelling (PLS-SEM) (Hair et al., 2014) to test our hypotheses. Consistent with the study’s objective to establish the predictive and nomological validity of FAIRFOOD, the PLS-SEM approach is preferable because of its focus on optimizing the prediction of endogenous constructs (Hair et al., 2017). In addition, PLS-SEM better serves exploratory and nomological purposes in situations of soft or limited theory (Hair et al., 2011; Sosik et al., 2009). Greater statistical power in PLS-SEM ensures that when a specific relationship exists in the population, it is more likely to be statistically significant (Hair et al., 2017). We followed a two-step procedure in analyzing and interpreting a research model (Hair et al., 2017), first analyzing the measurement model and then evaluating the structural model.

#### Confirmatory Factor Analysis of FAIRFOOD

Prior to testing our measurement and structural models in PLS-SEM, we performed a CFA on FAIRFOOD. Replicating the findings of Study 2, the second-order factorial model exhibited a good fit to the data for Italy (*χ*^2^ (73) = 137.64, CFI = 0.97, TLI = 0.97, RMSEA = 0.05, SRMR = 0.03) and the UK (*χ*^2^ (73) = 116.12, CFI = 0.98, TLI = 0.98, RMSEA = 0.04, SRMR = 0.04). The second-order factor loadings were statistically significant at 0.001 and substantive in size, ranging from 0.77 to 0.98 (*M* = 0.91) in Italy, and from 0.65 to 0.93 in the UK (*M* = 0.84). The measurement demonstrated good reliability (Italy: *ω* = 0.92; UK: *ω* = 0.87). The AVE for the second-order construct was 0.83 and 0.72 in the Italian and the UK samples, respectively. The measurement invariance tests support the configural, metric and scalar invariance of FAIRFOOD across the Italy and UK samples (see Web Appendix).

#### Measurement Model Assessment and Common Method *Bias*

We then evaluated the measurement model in PLS-SEM by examining reliability, convergent and discriminant validity of the latent constructs. All latent constructs were modelled as reflective. We used a repeated indicator approach to obtain parameter estimates for a second-order reflective construct of FAIRFOOD (Sarstedt et al., 2019).

Table 9 reports the measurement model assessment. We removed one item in the Italian sample and two items in the UK sample because their indicator loadings were below 0.50. In the UK sample, we also removed one item with the lowest indicator loading (0.51) from the Willingness to Purchase scale to ensure that the AVE for that latent construct was above 0.50. In both Italian and the UK samples, all the remaining indicators’ loadings were in the acceptable range and were significant at the 0.001 level, hence supporting the measurements’ convergent validity at the item level. Composite reliabilities for all latent variables in both samples were well above the recommended 0.70 cut-off value (Nunnally & Bernstein, 1994), demonstrating the reliability of the measurements. AVE values for all constructs in both samples exceeded the threshold of 0.50, indicating that each latent construct accounted for at least 50% of the variance in the items. We tested the discriminant validity of the latent constructs using the HTMT approach. The results in Table 9 indicate that the measurement model demonstrated sufficient discriminant validity. Finally, the results of the marker variable approach indicated no issues with common method bias in both samples (see Web Appendix).

**Table 9:** Psychometric properties of the measurements model (Study 4)

Latent construct	Adapted from	Item code	Italy			UK		
			λ	CR	AVE	Λ	CR	AVE
*First-order constructs*								
Commitment	Eisingerich & Rubera, (2010)	CMT 1	0.85	0.90	0.75	0.89	0.91	0.79
		CMT 2	0.90			0.87		
		CMT 3	0.80			0.90		
		CMT 5	0.90			0.90		
Experience of positive emotions	De Raad & Kokkonen (2000) and Snippe et al., (2018)	EPE 1	0.90	0.89	0.82	0.91	0.92	0.86
		EPE 2	0.92			0.94		
		EPE 3	0.90			0.93		
Experience of negative emotions	De Raad & Kokkonen, (2000) and Watson et al., (1988)	EME 1	0.93	0.92	0.84	0.94	0.94	0.88
		EME 2	0.90			0.95		
		EME 3	0.92			0.93		
Frequency of engagement	Habashi et al., (2016)	FRE 1	0.85	0.76	0.54	0.82	0.75	0.54
		FRE 2	0.74			0.80		
		FRE 3	0.66			0.67		
		FRE 4	0.67			0.63		
Willingness to purchase	Balabanis & Diamantopoulos, (2016), Giampietri et al., (2016), Zerbini et al., (2019)	WTP 1	0.72	0.85	0.51	0.84	0.79	0.53
		WTP 2	0.77			0.80		
		WTP 3	0.67			0.71		
		WTP 4	0.68			–		
		WTP 5	0.75			0.53		
		WTP 6	0.63			–		
		WTP 7	0.79			0.73		
*Second-order construct*								
FAIRFOOD		Social	0.90	0.94	0.80	0.85	0.91	0.71
		Economic	0.94			0.91		
		Informational	0.80			0.72		
		Environmental	0.92			0.89		

*Heterotrait-Monotrait ratio (HTMT)*						
	1	2	3	4	5	6
1. FAIRFOOD	–	0.50	0.31	0.45	0.32	0.42
2. Commitment	0.50	–	0.53	0.56	0.72	0.76
3. Experience of negative emotions	0.33	0.51	–	0.45	0.32	0.47
4. Experience of positive emotions	0.39	0.58	0.47	–	0.44	0.50
5. Frequency of engagement	0.33	0.71	0.42	0.49	–	0.73
6. Willingness to purchase	0.48	0.68	0.30	0.51	0.60	–

For FAIRFOOD, second-order standardized loadings are reported. CMT 4 was removed in both samples; WTP 4 and WTP 5 were removed in the UK sample. HTMT values for the Italian sample are reported below the diagonal and for the UK sample above the diagonal

*λ* standardized loadings, *FAIRFOOD* consumers’ disposition toward fairness in food supply chains

All loadings are significant at *p* < 0.001 level

#### Structural Model Assessment and Hypotheses Testing (Direct Effects)

After demonstrating that the measurement model exhibited satisfactory reliability and validity, we proceeded with the analysis of the structural model, to test the hypothesized relationships between the latent constructs (Henseler et al., 2009). We assessed the ability of the structural model to predict the endogenous latent variables (Hair et al., 2014) by examining the coefficient of determination (*R*^2^), the coefficient of predictive relevance (*Q*^2^ criterion), direction and significance of the path coefficients, and the effect size (*f*^2^). In addition, to examine the out-of-sample prediction power, we used PLSpredict. We calculated the statistical significance of the path coefficients using 5,000 bootstrapping samples. Prior to the main analysis, we assessed Variance Inflation Factors (VIFs) which were smaller than 2.0 in both samples, indicating that multicollinearity was not a serious concern.

**Fig. 2:**
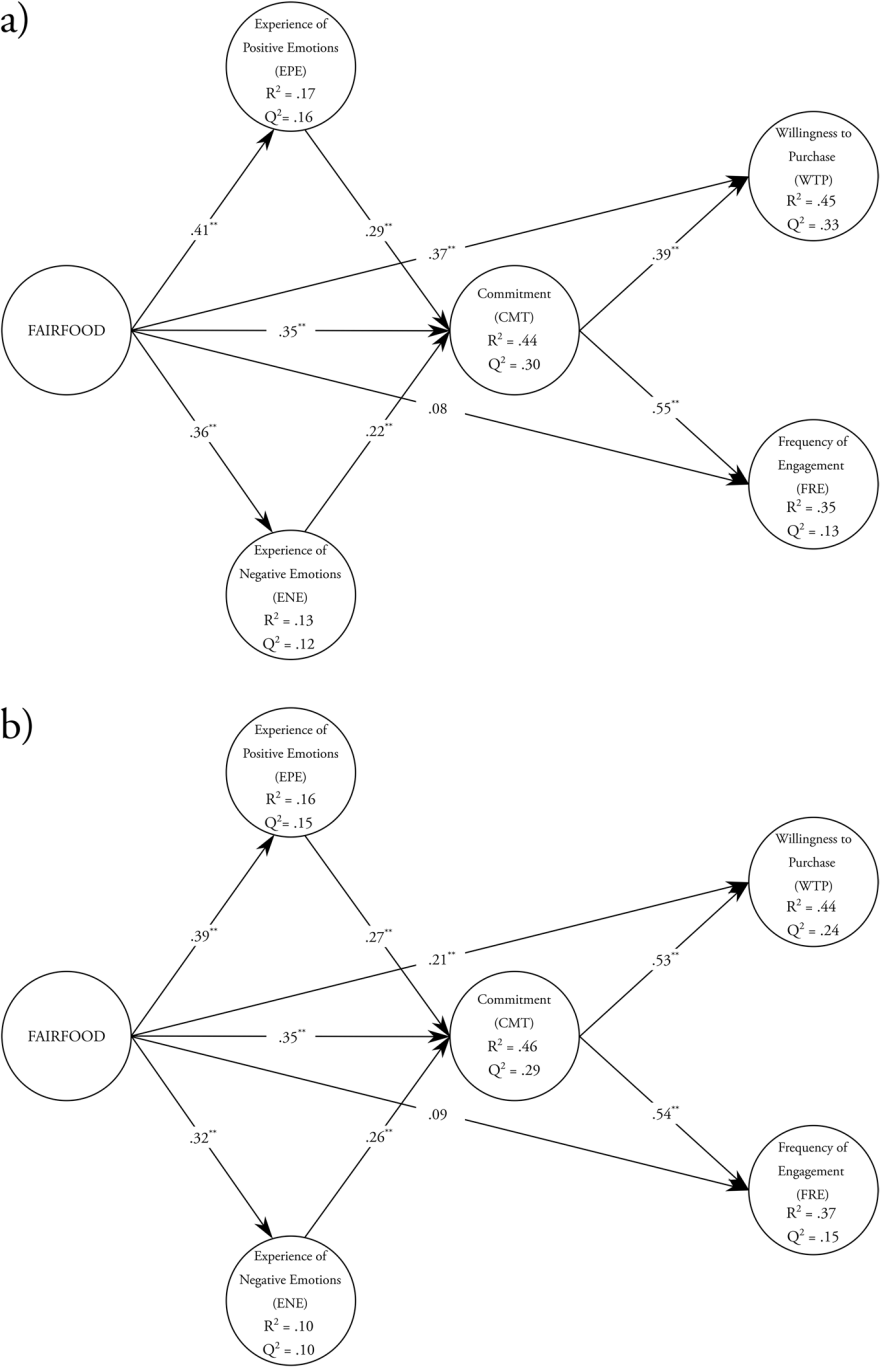
**a** Results for the hypothesized model for the Italian sample. **b** Results for the hypothesized model for the UK sample. FAIRFOOD = consumers’ disposition toward fairness in food supply chains. Standardized path estimates are reported. *R*^2^ and *Q*^2^ are given for endogenous constructs; **p < 0.001

As demonstrated in Fig. 2a, b, the *R*^2^ values of the endogenous latent variables ranged from 0.13 to 0.45 in the Italian and from 0.10 to 0.46 in the UK samples suggesting that the overall model exhibited adequate in-sample predictive power (e.g., Hair et al., 2011). In both samples, *Q*^*2*^ for endogenous constructs were well above zero, indicating that exogenous constructs had strong predictive relevance regarding the endogenous constructs (Hair et al., 2011). Next, we used the PLSpredict procedure (ten folds, ten repetitions; see Web Appendix) and RMSE as a prediction statistic to examine the model’s out-of-sample predictive power (Shmueli et al., 2019). For Italy, the majority of the dependent constructs’ indicators had lower prediction errors (in terms of RMSE) compared to the predictions generated for the indicators by the linear regression model (LM). Therefore, in the Italian sample the model exhibited medium predictive power (Shmueli et al., 2019). In the UK sample, a minority of the construct’s indicators produced lower prediction errors compared to the LM, meaning that the model had low predictive power.

Table 10 provides standardized path coefficients (*β*), significant levels (*t*-statics), and effect size (*f*^2^) for each path. The *f*^2^ values of 0.02, 0.15 and 0.35 indicate small, medium, and large effect sizes, respectively (Hedges, 1982). In both the Italian and the UK samples, the model path estimates revealed that FAIRFOOD has a positive direct effect on willingness to purchase FT certified products (Italy: *β* = 0.37, *p* < 0.001, *f*^2^ = 0.18; UK: *β* = 0.21, *p* < 0.001, *f*^2^ = 0.06) and commitment towards certified FT products (Italy: *β* = 0.35, *p* < 0.001, *f*^2^ = 0.18; UK: *β* = 0.35, *p* < 0.001, *f*^2^ = 0.18). Therefore, the results provide support for H1 and H2 respectively. The direct effect of FAIRFOOD on frequency of engagement with FT products was non-significant (Italy: *β* = 0.08, *p* = 0.18; UK: *β* = 0.09, *p* = 0.06). Therefore, H3 was not supported. In support of H4, FAIRFOOD has a significant and positive impact on the experience of positive emotions during the consumption of FT certified products (Italy: *β* = 0.41, *p* < 0.001, *f*^2^ = 0.20; UK *β* = 0.39, *p* < 0.001, *f*^2^ = 0.18). In H5 we predicted that dispositional fairness increases the experience of negative emotions when not engaging in consumption of FT products. This hypothesis received support in the Italian (*β* = 0.36, *p* < 0.001, *f*^2^ = 0.15) and the UK (*β* = 0.32, *p* < 0.001, *f*^2^ = 0.12) samples.

**Table 10:** Hypothesis assessment (Study 4)

Hypothesis		*β*	*t*-Value	*f*^*2*^	Decision
Italy					
*Direct effect*					
H1	FAIRFOOD → Willingness to purchase	0.37**	5.58	0.18	Supported
H2	FAIRFOOD → Commitment	0.35**	6.93	0.18	Supported
H3	FAIRFOOD → Frequency of engagement	0.08^n.s.^	1.34	0.01	Rejected
H4	FAIRFOOD → Experience of positive emotions	0.41**	8.65	0.20	Supported
H5	FAIRFOOD → Experience of negative emotions	0.36**	8.18	0.15	Supported
*Specific indirect effects*					
H6	FAIRFOOD → Commitment → Willingness to purchase	0.14**	5.37		Supported
H7	FAIRFOOD → Commitment → Frequency of engagement	0.19**	5.75		Supported
H8	FAIRFOOD → Experience of positive emotions → Commitment	0.12**	4.68		Supported
H9	FAIRFOOD → Experience of negative emotions → Commitment	0.08**	3.87		Supported
UK					
*Direct effect*					
H1	FAIRFOOD → Willingness to purchase	0.21**	3.64	0.06	Supported
H2	FAIRFOOD → Commitment	0.35**	7.65	0.18	Supported
H3	FAIRFOOD → Frequency of engagement	0.09^n.s.^	1.90	0.01	Rejected
H4	FAIRFOOD → Experience of positive emotions	0.39**	7.35	0.18	Supported
H5	FAIRFOOD → Experience of negative emotions	0.32**	6.37	0.12	Supported
*Specific indirect effects*					
H6	FAIRFOOD → Commitment → Willingness to purchase	0.19**	6.09		Supported
H7	FAIRFOOD → Commitment → Frequency of engagement	0.19**	6.24		Supported
H8	FAIRFOOD → Experience of positive emotions → Commitment	0.11**	4.57		Supported
H9	FAIRFOOD → Experience of negative emotions → Commitment	0.09**	4.18		Supported

*FAIRFOOD* consumers’ disposition toward fairness in food supply chains

Standardized estimates (*β*) are reported; *f*^2^is reported for direct effects. Critical *t*-values: **2.58 (*p* <  0.01); ^n.s.^ < 1.96 (*p* > 0.05)

#### Tests for Mediation (Indirect Effects)

Consistent with the non-parametric PLS path modeling approach, we used a non-parametric bootstrapping procedure to test the significance of the mediating effects (Henseler et al., 2009). As shown in Table 10, the results provide evidence for the hypothesized mediating roles of commitment towards FT products, the experience of positive emotions when consuming FT products, and the experience of negative emotions in the absence of FT product consumption. Specifically, in support of H6, the results indicate a positive and significant indirect effect of FAIRFOOD on willingness to purchase FT products via commitment (Italy: *β* = 0.14, *p* < 0.001; UK: *β* = 0.19, *p* < 0.001). Consequently, this result implies partial mediation because both the direct and indirect effects are significant. In support of H7, the indirect effect of FAIRFOOD on frequency of engagement with FT products via commitment is positive and significant (Italy: *β* = 0.19, *p* < 0.001; UK: *β* = 0.19, *p* < 0.001). Given that in both samples the direct effect is not significant while the indirect effect is significant, leads us to conclude that commitment fully mediates the relationship between FAIRFOOD and frequency of engagement.

In support of H8, the results of the mediation analysis reveal that there is a significant indirect effect of FAIRFOOD on commitment towards FT products via the experience of positive emotions during the consumption of certified FT products (Italy: *β* = 0.12, *p* < 0.001; UK: *β* = 0.11, *p* < 0.001). Since both the indirect and direct effects are significant, we conclude that the effect of FAIRFOOD on commitment is partially mediated by the experience of positive emotions. Further, in support of H9 the influence of FAIRFOOD on commitment towards FT products is partially mediated by the experience of negative emotions in the absence of consuming FT products, because both the direct and indirect effects (Italy: *β* = 0.08, *p* < 0.001; UK: *β* = 0.09, *p* < 0.001) are positive and significant.

## Discussion

Drawing on Fairness Theory this paper defines and conceptualizes consumers’ dispositions towards fairness in agri-food supply chains. Based on this, we developed a 14-item, multidimensional measurement tool (FAIRFOOD), utilizing a rigorous scale development process that included four studies over two countries (Italy and the UK) with eight independent samples. Across both countries, which represent very different food cultures, we find consistently high convergent and discriminant validity of the scale, and demonstrate its unique position in a nomological network of related ethical and marketing constructs. The last two studies provide evidence of predictive validity, helping uncover the mechanisms by which consumers’ dispositions toward fairness affect behavioral (e.g., purchase, boycott, WOM) intentions. Overall, the scale satisfies all criteria for newly developed construct measures (Böttger et al., 2017; DeVellis, 2016), and the paper offers novel insights for consumer ethics theory.

Fairness in the agri-food sector has become increasingly important to consumers, policy makers and agri-food industry practitioners (European Commission, 2020), with consumers’ and citizens’ preferences are driving increased regulation of food supply chains. Yet, despite growing attention from stakeholders, the conceptualization of fairness in food supply chains has been underdeveloped and FAIRFOOD is timely in addressing this gap. While previous measures of fairness exist, these often relate to a single dimension such as price fairness (Malc et al., 2016), consequently providing only a partial understanding of consumers dispositions toward fairness. FAIRFOOD emerges as distinct from related constructs like ethical consumption, FT concern, and pro-environment behavior, indicating its unique contribution to understanding the multidimensionality of fairness in agri-food systems.

### Theoretical Implications

First, we advance the literature by defining and conceptualizing consumer dispositions toward fairness in agri-food supply chains. Informed by Fairness Theory (Broome, 1990; Folger & Cropanzano, 2001), we recognise that fairness encompasses claims regarding the nature and distribution of outcomes, rights and constraints on different actors, and duties and obligations to others. In the context of agri-food supply chains, we demonstrate that a consumer’s disposition toward fairness has four dimensions—economic (Briggeman & Lusk, 2011), environmental (Abramovich & Vasiliu, 2022; Kilbourne & Pickett, 2008), social (Nickel, 2023; Sudbury-Riley & Kohlbacher, 2016; Toti et al., 2021), and informational (Greenberg, 1990; Smith et al., 2010) fairness. Recognizing that these dimensions are manifestations of the same construct, the results from our scale development process confirm and replicate the higher-order structure, comprising four dimensions over several samples and two cultures (Italy vs. the UK). The research thus provides a novel, holistic and robust conceptualization of consumers’ dispositions toward fairness in agri-food supply chains.

Secondly, the scale development process validates the conceptualization of fairness. Convergent and discriminant validity were demonstrated through the relation between FAIRFOOD and a plethora of constructs including ethical consumption behavior, justice, FT concern and skepticism, environmental belief, and concern. However, FAIRFOOD is empirically distinct from these variables as indicated by HTMT and Fornell & Larcker, (1981) tests for construct distinctiveness. FAIRFOOD thus fills an important gap in developing a multi-dimensional scale of consumers’ dispositions toward fairness in agri-food supply chains.

Thirdly, Studies 3 and 4 improve our understanding of the antecedents and consequences of consumers’ dispositions toward fairness in agri-food supply chains. Consumer activism—public demonstrations of support or opposition in response to the actions of others—is of increasing importance to marketers and managers (Moorman, 2020). We find that those with a higher disposition towards fairness in agri-food supply chains are more likely to commit to boycotting a brand they currently buy and spreading negative WOM about it if the brand is revealed to have treated other actors in the supply chain unfairly. Fairness also affects purchase intentions—those with a higher disposition towards fairness in agri-food supply chains are more willing to purchase, engage and make commitments toward FT certified products. Moreover, we explore the mechanisms by which consumers’ dispositions to fairness influence purchase intentions, drawing on trait theory. Specifically, we present evidence regarding how the relationships between the disposition towards fairness and willingness to purchase and frequency of engagement with FT products are partially and fully mediated by commitment, respectively. These results demonstrate that a disposition towards fairness enhances consumers’ commitment and encourages them to spend energy, time, and efforts to engage in the consumption of FT products. These findings are in keeping with consistency-based models of ethical decision making (Heger & Slonim, 2022). Consequently, consumers “adopt behavioral strategies that are consistent with their existing self-conceptions” (Escalas & Bettman, 2003, p. 340), so that those who place a strong emphasis on fairness in the agri-food setting (i.e., those high on FAIRFOOD) tend to maintain and reaffirm these self-views by committing and engaging in the consumption of disposition consistent products. Such consumption behaviors minimize inconsistency between how one currently perceives oneself and how one desires to view oneself (Higgins, 1987).

Finally, we contribute to and extend the literature concerning the relationship between fairness and consumer emotions (Malc et al., 2016). We expound that consumers with a stronger disposition towards fairness tend to experience more positive emotions when engaging in the consumption of FT certified products and feel more negative emotions in the absence of such consumption. Moreover, the experience of positive emotions during the consumption of FT products partially mediates the relationship between FAIRFOOD and commitment. These results indicate that a disposition towards fairness facilitates a positive emotional experience from consumption, which then heightens commitment towards FT consumption. These findings are consistent with theories of affect regulation (Cohen et al., 2008) as people strive to intensify, maintain, or prolong their positive affective states and attenuate negative states. By engaging in habitual consumption of products congruent with the notion of fairness, those high on the disposition towards fairness intensify and prolong their positive emotional states. For the same individuals, disengagement from FT consumption is a negative emotional experience, so that they are more likely to commit to FT consumption as the means to minimize the occurrence of negative feelings (e.g., Cohen & Andrade, 2004).

### Practical Implications

Globally, agri-food chains are becoming increasingly regulated to ensure fairer outcomes (European Commission, 2020), a process driven by consumer concerns. Understanding how consumers conceptualize fairness is thus increasingly important for food industry managers seeking to understand the dynamics of their industry. The lack of prior common understanding and conceptualization (Barling et al., 2022), could result in inconsistent or partial evaluations of fairness within the food system, leading to misunderstandings and a lack of trust between stakeholders, including consumers, producers, and retailers. To address this weakness, we provide practitioners with a clear conceptualization of FAIRFOOD and its underlying dimensions.

Regarding business strategy, a plethora of separate initiatives focus on specific dimensions of fairness, for instance, concentrating on improved environmental outcomes or returns to producers (Asioli et al., 2020). However, the scale development process indicates that economic, environmental, social, and informational fairness are dimensions of a higher-order structure. Rather than focusing on one dimension of fairness independently, to meet consumers’ expectations, managers should adopt a holistic approach, devising initiatives that address all four dimensions in tandem. In the absence of FAIRFOOD, managers may overlook the interrelationships between the economic, environmental, social, and informational dimensions of fairness, limiting their understanding and potentially leading to ill-conceived strategies and a misallocation of resources.

Initiatives that improve fairness within agri-food supply chains often have a higher underlying cost structure (Back et al., 2019). Managers consequently seek to identify consumers that are willing to pay a premium for products that lead to fairer agri-food supply chain outcomes (Bürgin & Wilken, 2022). Geographical, demographic, and social-economic variations in fairness dispositions may thus be useful in identifying the most promising locations and segments for initiatives, with areas and groups scoring higher on FAIRFOOD being more appealing. Aside from market segmentation (Ross & Milne, 2021), managers could also use the FAIRFOOD scale to investigate the salience of fairness in agri-food supply chains to their current and potential customers, as well as those of rivals. This can improve a company’s market intelligence regarding appropriate market positioning. As a parsimonious measure, FAIRFOOD can be readily and easily applied in market research.

Finally, the FAIRFOOD scale is relevant for policy makers, who recognize that the outcomes of their efforts to improve fairness in agri-food supply chains depend, in part, on consumer support (European Commission, 2020). In the absence of a comprehensive framework, policymakers may struggle to design effective regulations and policies that reflect the fairness concerns of consumers. This could hinder efforts to address pressing issues such as the sustainability of food systems. Integrating the FAIRFOOD scale into longitudinal citizen polling, such as Eurobarometer studies (European Commission, 2022), would provide the means to track changes in consumer dispositions toward fairness and assess relations with support for ‘Farm to Fork’ policies. Moreover, scales can also be useful in assessing the effectiveness of interventions. For example, several NGOs and public bodies seek, through education, to alter citizens’ awareness and dispositions toward fairness in agri-food supply chains (Vasileva & Reynaud, 2021). In such cases, the FAIRFOOD scale could measure changes over time and between control and treatment groups.

### Limitations and Future Research

This study is not without limitations, which can be addressed in future research. Our results support the construct validity of our measurement of dispositional fairness, yet we recognize that construct validity is never accomplished in a single study and that future research should replicate results across other samples, contexts, and study designs. Furthermore, due to the cross-sectional nature of our studies, we cannot confirm causal relationships between the constructs in the examined nomological network. Although it is theoretically plausible that dispositional fairness should influence the outcomes in our model, if causality and directionality were to be inferred, an alternative research design should be employed. We recognize that survey work may not be the most appropriate approach for capturing emotional responses. Moreover, future longitudinal studies utilizing FAIRFOOD could, for example, uncover whether a heightened disposition towards fairness occurs after individuals reflect on their past consumption decisions, lifestyle choices, and marketplace experiences.

Another limitation pertains to the external validity and generalizability of our findings. Despite validating FAIRFOOD and replicating our results in two contexts with contrasting food cultures (Italy and the UK), our psychometric validation efforts are still restricted to Western European societies. Although the concept of a disposition towards fairness is likely to be universal, its manifestations might differ based on cultural differences (Berman et al., 1985; Kim & Leung, 2007). Forces often prominent in non-Western cultures, such as stronger collectivist tendencies, might influence the etiology of dispositional fairness (Bolton et al., 2003), altering the underlying factor structure of the superordinate construct. Therefore, further examination of measurement invariance and the factor structure of FAIRFOOD across various cultural contexts is necessary to confirm that the economic, social, environmental, and informational dimensions function as specific manifestations of a disposition towards fairness in agri-food chains. For instance, validation in South America (relatively high collectivist and power distance societies) is warranted.

Future enquiries could examine additional antecedents, such as legal requirements and regulations (Kaplan & Ferris, 2001), which may explain a consumer's disposition towards fairness. Moreover, while not a focus of this paper, regression analysis in Study 3 identifies discrepancies between the samples for Italy and the UK regarding relations between moral identity, personality traits, and fairness dispositions. Consequently, further research examining how the correlates, outcomes and antecedents of fairness dispositions vary across cultures is warranted. Furthermore, researchers could investigate other mediating mechanisms (e.g., affective, cognitive, and motivational) underlying the impact of dispositional fairness on commitment, willingness to purchase, and engagement with FT products.

Finally, one could also use FAIRFOOD to examine the role of dispositions towards fairness and self-oriented or egoistic motivations in ethical consumption. Our results support the notion that individuals with high FAIRFOOD scores are likely to be other-oriented in their decision-making. However, consumers' ethical consumption can also be driven by self-oriented or egoistic motives, such as self-actualization and narcissism, alongside altruism (Hwang & Kim, 2018). Consequently, future research could examine the relationship between FAIRFOOD and various egoistic motivations and self-orientation factors, such as self-actualization (Hwang & Kim, 2018) and egoistic self-interest (Cialdini et al., 1987). Additionally, one could investigate the factors that trigger altruistic or egoistic motivations in specific purchase situations among individuals scoring differently on FAIRFOOD. Researchers could also examine whether marketing messages based on other-benefit (altruistic) versus self-benefit (egoistic) appeals (White & Peloza, 2009) are more effective for individuals scoring highly (vs. lowly) on FAIRFOOD. Additionally, future research could explore the circumstances (e.g., private vs. public consumption) under which each type of appeal is most persuasive to high and low FAIRFOOD scoring consumers.

## Supplementary Information

Below is the link to the electronic supplementary material.

Supplementary file1 (DOCX 96 KB)

## Data Availability

For inquiries regarding data availability, please contact the authors.

## Footnotes

1 In the UK sample, the difference between the original model and the three-factorial model in which social and economic dimensions were merged together was non-significant (Δ*χ*^2^ [1] = 0.327, *p* = 0.57). However, given that (1) there was no substantial improvement in the model fit based on ΔCFI and ΔRMSEA, (2) significant Δ*χ*2 tests for these models in Italy (Δ*χ*^2^ [1] = 18.29, *p* < 0.001), and (3) theoretical accounts, we retained the second-order, four-dimensional operationalisation.

2 Although our main goal was to provide evidence for discriminant validity of FAIRFOOD, we note that some of the studied constructs exhibit marginal issues with discriminant validity. Among the three constructs of FTO justice, discriminant validity could be deemed only when using a more liberal critical value (0.90) for HTMT2.
