# Supplementary material for: Consumer Disposition Toward Fairness in Agri-Food Chains (FAIRFOOD): Scale Development and Validation
Source: J Bus Ethics. 2024 Jul 19;197(2):391–421. doi: 10.1007/s10551-024-05756-2 (PMC11893701; doi:10.1007/s10551-024-05756-2)
Supplement: Supplementary file 1 — Supplementary file1 (DOCX 96 KB) [file 10551_2024_5756_MOESM1_ESM.docx]

CONSUMER DISPOSITION TOWARD FAIRNESS IN THE AGRI-FOOD CHAIN (FAIRFOOD): SCALE DEVELOPMENT AND VALIDATION

[WEB] APPENDIX

**Table 1** Study 1 - Literature sources of the 42 questionnaire items

| ***Items*** | **References** |
| --- | --- |
| *Social dimension* |  |
| 1. Guarantee no discrimination | (Fairtrade, 2022; Food Ethics Council, 2020; Nickel 2023; Greenberg 1990; European Parliament 2021; Tian et al. 2021; Toti et al. 2021; Sudbury-Riley and Kohlbacher 2016) |
| 1. Avoid agro-mafia (gangmaster) | (Fairtrade, 2022; Nickel 2023; Greenberg 1990; European Parliament 2021; Tian et al. 2021; Toti et al. 2021; Sudbury-Riley and Kohlbacher 2016) |
| 1. Avoid child labour | (FAO, 2022; Cho et al., 2019; Fairtrade, 2022; Nickel 2023; Greenberg 1990; European Parliament 2021; Tian et al. 2021; Toti et al. 2021; Sudbury-Riley and Kohlbacher 2016) |
| 1. Guarantee training opportunities to workers | (Fairtrade, 2022; Food Ethics Council, 2020; Nickel 2023; Tian et al. 2021; Toti et al. 2021; Sudbury-Riley and Kohlbacher 2016) |
| 1. Include small scale producers | (Chang & Lusk, 2009; FAO, 2022; Fairtrade, 2022) |
| 1. Provide local products | (Czeczotko et al., 2021; Hoang, 2021; Winterstein & Habisch, 2021) |
| 1. Include disadvantaged people (immigrants, disabled, etc.) | (Fairtrade, 2022; Food Ethics Council, 2020; European Parliament 2021; Toti et al. 2021; Sudbury-Riley and Kohlbacher 2016) |
| 1. Promote traditional products | (Fairtrade, 2022; Food Ethics Council, 2020) |
| 1. Ensure activities that do not require excessive physical exertion and respecting normal life times | (Food Ethics Council, 2020; Nickel 2023; Greenberg 1990; European Parliament 2021; Tian et al. 2021; Toti et al. 2021; Sudbury-Riley and Kohlbacher 2016) |
| *Economic dimension* |  |
| 1. Guarantee producers a remuneration that covers production costs | (Adams, 1963; Fairtrade, 2022; Food Ethics Council, 2020; Gielissen & Graafland, 2009 Samoggia et al. 2021; European Commission 2021) |
| 1. Charge the same price for organic and conventional product | (IFOAM website) |
| 1. Ensure good value for money | (Bolton et al. 2003; Diller 2008; Nguyen & Klaus, 2013; De Pelsmacker and Janssens 2007; Shih-Tse Wang and Chen 2019) |
| 1. Invest in supply chain innovation projects | (Fairtrade, 2022) |
| 1. Invest in projects in the community's interest | (Fairtrade, 2022) |
| 1. Guarantee producers stronger relationships with buyers | (Fairtrade, 2022; Samoggia et al. 2021; Briggeman and Lusk 2011; European Commission 2021) |
| 1. Make consumers pay a higher price in order to ensure fair pay for the actors in the chain | (Jeong et al., 2021, Busch & Spiller, 2016; Gielissen & Graafland, 2009; Gudbrandsdottir et al. 2021; Briggeman and Lusk 2011; European Commission 2021; Bolton et al. 2003; Diller 2008) |
| 1. Make food retailers ensure farmers receive a fair price for their agricultural products | (Busch & Spiller, 2016; Gielissen & Graafland, 2009; Gudbrandsdottir et al. 2021; Briggeman and Lusk 2011; European Commission 2021) |
| 1. Make food processors ensure farmers receive a fair price for their agricultural products | (Busch & Spiller, 2016; Gielissen & Graafland, 2009; Gudbrandsdottir et al. 2021; Briggeman and Lusk 2011; European Commission 2021) |
| 1. Make policies promote an information campaign for farmers to receive a fair price | (Busch & Spiller, 2016; Gielissen & Graafland, 2009; Gudbrandsdottir et al. 2021; Briggeman and Lusk 2011; European Commission 2021) |
| 1. Make policies ensure farmers receive a fair price for their agricultural products | (Busch & Spiller, 2016; Gudbrandsdottir et al. 2021 Samoggia et al. 2021; Briggeman and Lusk 2011; European Commission 2021) |
| 1. Have a low price for consumers | (Bolton et al. 2003; Diller 2008; Nguyen & Klaus, 2013; Shih-Tse Wang and Chen 2019) |
| *Informational dimension* |  |
| 1. Indicate the origin of the ingredients | (Food Ethics Council, 2020; Aprile, Caputo, & Nayga, 2012; Greenberg 1990; European Parliament 2011) |
| 1. Highlight the expiration date of the products | (Food Ethics Council, 2020; Smith et al. 2010; European Parliament 2011) |
| 1. Indicate the cultivation and breeding methods | (Food Ethics Council, 2020; Smith et al. 2010; European Parliament 2011) |
| 1. Use labels, standards and certifications | (Brenton, 2018; Nguyen & Klaus, 2013; Zepeda, Sirieix, Pizarro, Corderre, & Rodier, 2013; Aprile, Caputo, & Nayga, 2012; Smith et al. 2010; European Parliament 2011; Phlips 1983) |
| 1. Sell tasty products | (De Pelsmaeker et al., 2017; Samoggia 2021) |
| 1. Indicate price distribution information on labels | (Food Ethics Council, 2020; Greenberg 1990; Smith et al. 2010; European Parliament 2011; De Pelsmacker and Janssens (2007) |
| 1. Have a discount for consumers | (Nguyen & Klaus, 2013) |
| 1. Ensure no waste | (Fairtrade, 2022; FAO, 2021) |
| 1. Promote "pick-your-own" option | (Hoang, 2021; Sacchi, 2018) |
| 1. Promote farm selling | (Hoang, 2021; Sacchi, 2018) |
| 1. Promote farmers market | (Hoang, 2021; Sacchi, 2018) |
| 1. Strengthen the direct relationship with producers | (Hoang, 2021; Sacchi, 2018; Greenberg 1990; Smith et al. 2010; European Parliament 2011; Phlips 1983) |
| *Environment dimension* |  |
| 1. Guarantee a natural product, with no modification of colour, shape or appearance for commercial purposes | (Czeczotko et al., 2021; Korzen et al., 2011; Rozin et al., 2004; Kilbourne and Pickett 2008; Fairtrade, 2022; FAO, 2021; Food Ethics Council, 2020) |
| 1. Promote easier access to nutritious food avoiding junk food | (FAO, 2021; Food Ethics Council, 2020) |
| 1. Guarantee healthy food (hormones free, antibiotics free, etc) | (Czeczotko et al., 2021; Konuk 2017, Korzen et al., 2011, Shaw et at., 2005, Rozin et al., 2004; Kilbourne and Pickett 2008; Fairtrade, 2022; FAO, 2021; Food Ethics Council, 2020) |
| 1. Guarantee animal welfare | (Beck & Ladwig, 2021; FAO, 2021; Reis et al., 2021; Food Ethics Council, 2020; Höglund, 2020; Grumett, 2019; Nawroth et al., 2019; Swaffield et al., 2019; Kilbourne and Pickett (2008) |
| 1. Be vegan | (Beck & Ladwig, 2021; Alvaro, 2017) |
| 1. Be organic | (Shaw et at., 2005, Chang & Lusk, 2009; Aprile, Caputo, & Nayga, 2012, Bartels & Onwezen, 2014, Konuk 2017, Czeczotko et al., 2021; Winterstein & Habisch, 2021; Golob et al. 2018; Thφgersen 1999; Toti et al. 2021) |
| 1. Include Fair Trade products | (Nguyen & Klaus, 2013; De Pelsmacker and Janssens 2007; Shih-Tse Wang and Chen 2019) |
| 1. Guarantee soil protection, biodiversity, water and waste management | (Czeczotko et al., 2021; De Olde & Valentinov, 2019; Fairtrade, 2022; FAO, 2021; Peano et al., 2019; Zimmerer et al., 2019; Abramovich and Vasiliu 2023) |
| 1. Guarantee sustainable packaging | (Golob et al. 2018; Thφgersen 1999; Sudbury-Riley and Kohlbacher 2016; FAO, 2021) |

***Supplement materials for Study 2***

Table 2. Measurements used in Study 2.

| Construct | Source/  Adapted from | Item | Response format |
| --- | --- | --- | --- |
| Fair trade concern | De Pelsmacker & Janssens (2007) | Fair trade is important | 1 = Strongly disagree;  7 = Strongly agree |
|  |  | Fair trade ought to be a generalized way of trading (the benchmark) and not an alternative way (the exception) |  |
|  |  | I am concerned about the fair trade issue |  |
| Fair trade scepticism | De Pelsmacker & Janssens (2007) | Fair trade is too much like charity: purchasing fair trade products does not solve anything in the long run. It just eases your conscience | 1 = Strongly disagree;  7 = Strongly agree |
|  |  | Fair trade products lack credibility |  |
|  |  | Fair trade makes me think of a colonial attitude by means of which we impose our norms to others |  |
| Fair trade information quality | De Pelsmacker & Janssens (2007) | The origin of fair trade products often cannot be traced | 1 = Strongly disagree;  7 = Strongly agree |
|  |  | Fair trade is a not well defined concept that should be explained concisely and clearly |  |
|  |  | There is only low-quality information about fair trade |  |
| Purchase intention toward fair trade products | Shih-Tse & Chen (2019) | I may consider purchasing fair trade products | 1 = Strongly disagree;  7 = Strongly agree |
|  |  | I intend to try fair trade products |  |
|  |  | I am interested in trying a fair trade product |  |
| Perceived distributive justice of FTOs | Shih-Tse & Chen (2019) | I believe that fair trade organisations ensure fair incomes for the producers (i.e., farmers and workers) of fair trade product | 1 = Strongly disagree;  7 = Strongly agree |
|  |  | I believe that fair trade organisations ensure the producers of fair trade products to receive fair returns |  |
|  |  | I believe that each producer receives a fair share of fair trade organisations resources |  |
| Perceived procedural justice of FTOs | Shih-Tse & Chen (2019) | I believe that fair trade organisations create policies and make decisions with the well-being of producers in mind | 1 = Strongly disagree;  7 = Strongly agree |
|  |  | I believe that fair trade organisations' procedures and guidelines are fair |  |
|  |  | I believe that fair trade organisations provide justice for producers according to the fair procedures |  |
| Perceived interactional justice of FTOs | Shih-Tse & Chen (2019) | I hold that fair trade organisations respect producers' rights | 1 = Strongly disagree;  7 = Strongly agree |
|  |  | I hold that fair trade organisations treat producers with dignity and respect |  |
|  |  | I believe that when problems arise or conflicts occur, fair trade organisations provide the producers with sufficient opportunities to make explanations |  |
| Environmental belief | Kilbourne & Pickett (2008) | Many types of pollution are rising to dangerous levels | 1 = Strongly disagree;  7 = Strongly agree |
|  |  | Some living things are being threatened with extinction |  |
|  |  | Continued use of chemicals in agriculture will damage the environment |  |
|  |  | Shortages of some important resources will occur in the near future |  |
| Environmental concern | Kilbourne & Pickett (2008) | I am very concerned about the environment | 1 = Strongly disagree;  7 = Strongly agree |
|  |  | Humans are severely abusing the environment |  |
|  |  | I would be willing to reduce my consumption to help protect the environment |  |
|  |  | Anti-pollution laws should be enforced more strongly |  |
| Ethical consumption | Toti et al. (2021) | I prefer buying products with an eco-label | 1 = Strongly disagree;  7 = Strongly agree |
|  |  | I prefer to buy in shops that feature ecological or organic products |  |
|  |  | I prefer to do my shopping in stores that promote fair trade |  |
|  |  | I buy fair-trade products to help producers |  |
|  |  | I buy products sold through social actions |  |
|  |  | I avoid brands/products that profit from the misery of their employees |  |
|  |  | I avoid products or brands that make children work even indirectly |  |
|  |  | I avoid products from companies that do not respect the rights of their employees |  |
|  |  | I restrict my consumption (food, energy, clothing, etc.) to what I really need |  |
|  |  | I contribute to the preservation of the environment through everyday actions |  |
|  |  | To reduce my contribution to global warming, I consume differently |  |
| Social media self-control failure | Du et al. (2018) | How often do you give in to a desire to use social media even though your social media use at that particular moment...^a^ | 1 = Almost never;  5 = Very often |
|  |  | ...conflicts with other goals (for example: doing things for school/study/work or other tasks)? |  |
|  |  | ...makes you use your time less efficiently? |  |
|  |  | ...makes you delay other things you want or need to do? |  |
| *Note*: ^a^ = item stem. | | | |

Table 3. Model comparison results from the CFA marker variable analysis (Study 2)

| Model | χ^2^ | *df* | CFI | Δχ^2^(Δdf) | Model Comparison |
| --- | --- | --- | --- | --- | --- |
| Italy | | | | | |
| CFA | 2157.01 | 1466 | .932 | — | — |
| Baseline | 2181.57 | 1483 | .931 | — | — |
| Method-C | 2176.41 | 1482 | .932 | 7.31 (1)^**^ | vs. Baseline |
| Method-U | 2105.29 | 1429 | .933 | 71.02 (53)^*^ | vs. Method-C |
| Method-R | 2094.79 | 1484 | .940 | 0.433 (55)^n.s.^ | vs. Method-U |
| U.K. | | | | | |
| CFA | 2451.02 | 1466 | .933 | — | — |
| Baseline | 2498.70 | 1483 | .931 | — | — |
| Method-C | 2489.72 | 1482 | .932 | 8.10 (1)^**^ | vs. Baseline |
| Method-U | 2370.00 | 1429 | .936 | 120.04 (53)^**^ | vs. Method-C |
| Method-R | 2364.73 | 1484 | .940 | 2.70 (55) ^n.s.^ | vs. Method-U |
| Note: Scaled χ2 and Δχ2 are reported. df = degrees of freedom.  ^**^ p < .001; ^*^ p < .05; ^n.s.^ = non-significant (*p* > .05) | | | | | |

Table 4. Model comparison (Study 2).

|  |  | Italy | | | |  | U.K. | | | | |
| --- | --- | --- | --- | --- | --- | --- | --- | --- | --- | --- | --- |
| Model | Merged dimensions | χ^2^ (df) | CFI | RMSEA | Δχ2(Δdf) |  | χ^2^ (df) | CFI | RMSEA | | Δχ2(Δdf) |
| 4 factors |  | 76.85 (73) | .998 | .013 |  |  | 168.07 (73) | .969 | .056 | |  |
| 3 factors | SOC and ECO | 115.75 (74) | .977 | .042 | 18.29(1)^**^ |  | 166.67 (74) | .969 | .055 | | 0.327 (1)^n.s.^ |
| 3 factors | SOC and INF | 277.24 (74) | .887 | .092 | 79.27 (1) ^**^ |  | 305.35 (74) | .924 | .086 | | 69.02 (1) ^**^ |
| 3 factors | SOC and ENV | 113.04 (74) | .978 | .040 | 14.20 (1)^**^ |  | 235.71 (74) | .947 | .072 | | 30.86 (1) ^**^ |
| 3 factors | ECO and INF | 246.59 (74) | .904 | .084 | 76.08 (1) ^**^ |  | 306.92 (74) | .923 | .087 | | 52.58(1) ^**^ |
| 3 factors | ECO and ENV | 135.87 (74) | .966 | .051 | 30.19 (1) ^**^ |  | 257.84 (74) | .939 | .077 | | 25.91 (1) ^**^ |
| 3 factors | INF and ENV | 252.55 (74) | .901 | .086 | 105.28 (1) ^**^ |  | 252.42 (74) | .941 | .076 | | 38.44 (1) ^**^ |
| 2 factors | SOC, ECO, and INF | 298.30 (75) | .876 | .095 | 81.40(2) ^**^ |  | 328.25 (75) | .916 | .090 | | 64.97(2) ^**^ |
| 2 factors | SOC, ECO, and ENV | 166.72 (75) | .949 | .061 | 36.37 (2) ^**^ |  | 283.29 (75) | .931 | .082 | | 42.57(2) ^**^ |
| 2 factors | SOC, INF, and ENV | 303.43 (75) | .873 | .097 | 84.84 (2) ^**^ |  | 347.77 (75) | .910 | .093 | | 71.44 (2)^**^ |
| 2 factors | ECO, INF, and ENV | 311.07 (75) | .869 | .098 | 93.84 (2) ^**^ |  | 375.21 (75) | .901 | .098 | | 66.97 (2) ^**^ |
| 1 factor | All | 349.36(77) | .849 | .104 | 152.44(4) ^**^ |  | 408.24 (77) | .891 | .101 | | 126.22 (4) ^**^ |
| *Note*: Comparisons are made with respect to the baseline model (4 factors). All models are modelled as second-order.  SOC = social, ECO = economical; INF = informational; ENV = environmental.  Scaled χ^2^ and Δχ^2^ are reported. *df* = degrees of freedom.  ^**^*p* < .001; ^n.s.^ = non-significant (*p* > .05). | | | | | | | | | |  |  |

**3. Supplement materials for Study 4**

| Table 5. Measurements used in Study 3. | | | |
| --- | --- | --- | --- |
| Construct | Source/  Adapted from | Item | Response format |
| Moral identity | Aquino & Reed (2002) | 1. It would make me feel good to be a person who has these characteristics. 2. Being someone who has these characteristics is an important part of who I am. 3. I would be ashamed to be a person who had these characteristics (R). 4. Having these characteristics is not really important to me. (R) 5. I strongly desire to have these characteristics. | 1 = Strongly disagree;  5 = Strongly agree |
| Self-Efficacy in Ethical Consumption. |  | 1. I'm confident in understanding ethical labels (like fair-trade, organic). 2. I can find ethical products, even if it's harder or takes longer. 3. I can get friends and family to choose ethical products with the information I provide. 4. I can prioritize buying from companies that I know are socially responsible. 5. I can easily find trustworthy information about the ethics of products. 6. I believe I can budget my shopping to include more ethical products, despite their price | 1 = Strongly disagree;  7 = Strongly agree |
| Ethical sensitivity | Toti & Moulins (2017) | 1. I am against injustice in all its forms. 2. I have respect for others (their culture, privacy, personality, etc.). 3. I am sensitive to the well-being of others. 4. It is important for me not to harm others. 5. The interests of others take precedence over my personal interests. 6. I consider the interests of others in my decision-making. 7. Gifts (of money, time, blood, objects, etc.) make me feel useful. 8. Volunteering is essential for the life of the community. | 1 = Strongly disagree;  7 = Strongly agree |
| Need for cognition | Cacioppo et al. (1984) | 1. I would prefer complex to simple problems. 2. I like to have the responsibility of handling a situation that requires a lot of thinking. 3. Thinking is not my idea of fun. (R) 4. I would rather do something that requires little thought than something that is sure to challenge my thinking abilities. (R) 5. I really enjoy a task that involves coming up with new solutions to problems. 6. I would prefer a task that is intellectual, difficult, and important to one that is somewhat important but does not require much thought. | 1 = Extrmely uncharacteristic of me;  5 = Extremely characteristic of me. |
| Social desirability | Vésteinsdóttir et al. (2017) | 1. I have never intensely disled anyone. 2. I sometimes feel resentful when I don’t get my way. (R) 3. No matter who I’m talking to, I’m always a good listener. 4. There have been occasions when I took advantage of someone. (R) 5. I’m always willing to admit it when I make a mistake. 6. I sometimes try to get even, rather than forgive and forget. (R) 7. There have been occasions when I felt like smashing things. (R) 8. There have been times when I was quite jealous of the good fortune of others. (R) 9. I have never felt that I was punished without cause. 10. I have never deliberately said something that hurt someone’s feelings. | 1 = Strongly disagree;  7 = Strongly agree |
| Conscientiousness | Maples-Keller et al. (2019) | I..   1. Handle tasks smoothly. 2. Know how to get things done. 3. Like to tidy up. 4. Leave a mess in my room. (R) 5. Tell the truth. 6. Break my promises. (R) 7. Work hard. 8. Set high standards for myself and others. 9. Carry out my plans 10. Have difficulty starting tasks. (R) 11. Make rash decisions. (R) 12. Act without thinking. (R) | 1 = Very innacuarate;  5 = Very accurate |
| Agreeableness | Maples-Keller et al. (2019) | I..   1. Trust others. 2. Believe that others have good intentions. 3. Cheat to get ahead. (R) 4. Take advantage of others. (R) 5. Love to help others. 6. Am concerned about others. 7. Insult people. (R) 8. Get back at others (R). 9. Believe that I am better than others. (R) 10. Think highly of myself. (R) 11. Sympathize with the homeless. 12. Feel sympathy for those who are worse off than myself. | 1 = Very innacuarate;  5 = Very accurate |
| Boycott intention | Klein et al. (2004); Lasarov et al. (2023)  Zarantonello et al. (2016) | 1. I would boycott the products of this brand. 2. I would stop purchasing products of this brand. 3. I would participate in boycotting this brand. | 1 = Strongly disagree;  7 = Strongly agree |
| Negative word-of-mouth intention | Klein et al. (2004), Wood & Karau (2009),  Zarantonello et al. (2016) | 1. I would spread negative word of mouth about this brand. 2. I would denigrate this brand to my friends. 3. When my friends were looking for a similar service/product, I would tell them not to buy from this brand. 4. I will tell my friends about my feelings towards this brand. 5. I would try to influence many people not to purchase from this brand. | 1 = Strongly disagree;  7 = Strongly agree |

*R* = reverse coded item. Social media self-control failure was used as a marker variable as in Study 3.

Table 6. Results of invariance tests (Study 3).

|  | Models | χ^2^ (*df*) | Δχ^2^(Δ*df*) | CFI | RMSEA | SRMR | ΔCFI | ΔRMSEA | ΔSRMR |
| --- | --- | --- | --- | --- | --- | --- | --- | --- | --- |
| **Italy vs. U.K.** |  |  |  |  |  |  |  |  |  |
| M1: Configural invariance | — | 227.82(146) | — | .976 | .044 | .042 | — | — | — |
| M2: Metric invariance of the first-order factors | M2: M1 | 254.22(156) | 26.86 (10)^*^ | .971 | .047 | .052 | .005 | .003 | .01 |
| M3: Metric invariance of the first- and second- order factors | M3: M2 | 255.47 (159) | 1.07 (3)^n.s.^ | .972 | .046 | .054 | .001 | .001 | .002 |
| M4: Scalar invariance of the first-order factors | M4: M3 | 317.77 (169) | 83.71(10)^**^ | .957 | .055 | .056 | .015 | .009 | .002 |
| M5: Scalar invariance of the first- and second- order factors | M5: M4 | 366.85 (172) | 59.41 (3)^**^ | .943 | .063 | .070 | .014 | .008 | .014 |
| **Italy: Male vs. Female** |  |  |  |  |  |  |  |  |  |
| M1: Configural invariance | — | 241.61 (146) | — | .948 | .069 | .060 | — | — | — |
| M2: Metric invariance of the first-order factors | M2: M1 | 257.65 (156) | 16.16 (10)^n.s.^ | .945 | .068 | .070 | .003 | .001 | .01 |
| M3: Metric invariance of the first- and second- order factors | M3: M2 | 261.96 (159) | 4.34 (3)^n.s.^ | .944 | .068 | .081 | .001 | .00 | .011 |
| M4: Scalar invariance of the first-order factors | M4: M3 | 276.50 (169) | 13.73 (10)^n.s.^ | .941 | .068 | .082 | .003 | .00 | .001 |
| M5: Scalar invariance of the first- and second- order factors | M5: M4 | 280.58 (172) | 3.85 (3)^n.s.^ | .941 | .067 | .084 | .00 | .001 | .002 |
| **U.K.: Male vs. Female** |  |  |  |  |  |  |  |  |  |
| M1: Configural invariance | — | 184.43 (146) | — | .983 | .043 | .040 | — | — | — |
| M2: Metric invariance of the first-order factors | M2: M1 | 194.20 (156) | 8.84 (10)^n.s.^ | .983 | .041 | .046 | .00 | .002 | .006 |
| M3: Metric invariance of the first- and second- order factors | M3: M2 | 198.89 (159) | 5.21 (3)^n.s.^ | .982 | .042 | .056 | .001 | .001 | .01 |
| M4: Scalar invariance of the first-order factors | M4: M3 | 214.70 (169) | 16.59 (10)^n.s.^ | .980 | .043 | .058 | .002 | .001 | .002 |
| M5: Scalar invariance of the first- and second- order factors | M5: M4 | 218.16 (172) | 3.35 (3)^n.s.^ | .980 | .043 | .058 | .00 | .00 | .00 |
| *Note*: Scaled χ^2^ and Δχ^2^ are reported. *df* = degrees of freedom.  ^*^ *p* < .05*;* ^**^*p* < .001; ^n.s.^ = non-significant (*p* > .05). | | | | | | | | | |

Table 7. Bivariate correaltions and marker adjusuted partial correaltions for the UK sample (Study 3).

|  | 1 | 2 | 3 | 4 | 5 | 6 | 7 | 8 | 9 | 10 | 11 |
| --- | --- | --- | --- | --- | --- | --- | --- | --- | --- | --- | --- |
| 1. FAIRFOOD |  | .39^***^ | .19^**^ | .57^***^ | .51^***^ | .15^*^ | .35^***^ | .57^***^ | .57^***^ | .17^**^ |  |
| 1. Moral identity | .39^***^ |  | .12^*^ | .18^**^ | .57^***^ | .22^***^ | .46^***^ | .29^***^ | .24^***^ | .24^***^ |  |
| 1. Need for cognition | .19^**^ | .12^*^ |  | .25^***^ | .21^***^ | .43^***^ | .12^*^ | .15^*^ | .12^*^ | .15^*^ |  |
| 1. Self-efficacy (EC) | .57^***^ | .18^**^ | .25^***^ |  | .40^***^ | .14^*^ | .24^***^ | .48^***^ | .45^***^ | .18^**^ |  |
| 1. Ethical sensitivity | .51^***^ | .57^***^ | .21^***^ | .40^***^ |  | .09 | .58^***^ | .37^***^ | .40^***^ | .23^***^ |  |
| 1. Conscientiousness | .15^**^ | .22^***^ | .43^***^ | .14^*^ | .10 |  | .18^**^ | .06 | .00 | .33^***^ |  |
| 1. Agreeableness | .35^***^ | .46^***^ | .12^*^ | .24^***^ | .58^***^ | .18^**^ |  | .23^***^ | .18^**^ | .52^***^ |  |
| 1. Boycott intention | .57^***^ | .29^***^ | .15^**^ | .48^***^ | .37^***^ | .06 | .23^***^ |  | .79^***^ | .03 |  |
| 1. Negative WOM | .57^***^ | .24^***^ | .12^*^ | .45^***^ | .40^***^ | .00 | .18^**^ | .79^***^ |  | -.01 |  |
| 1. Social desirability | .17^**^ | .24^***^ | .15^**^ | .18^**^ | .23^***^ | .33^***^ | .52^***^ | .03 | -.01 |  |  |
| 1. Marker | .00 | .13^*^ | -.21** | -.04 | .20^***^ | -.36^***^ | .00 | .02 | .05 | -.24^***^ |  |

*Note*: Correlations adjusted for the smallest correaltion with the marker are reported above the diagonal. FAIRFOOD = consumers’ disposition toward fairness in food supply chains

^***^ *p* < .001; ^**^*p* < .01; ^*^ *p* < .05*;*

Table 8. Bivariate correaltions and marker adjusuted partial correaltions for the Italian sample (Study 3).

|  | 1 | 2 | 3 | 4 | 5 | 6 | 7 | 8 | 9 | 10 | 11 |
| --- | --- | --- | --- | --- | --- | --- | --- | --- | --- | --- | --- |
| 1. FAIRFOOD |  | .37^***^ | .21^***^ | .39^***^ | .51^***^ | .30^***^ | .38^***^ | .46^***^ | .46^***^ | .12^*^ |  |
| 1. Moral identity | .37^***^ |  | .24^***^ | .29^***^ | .52^***^ | .39^***^ | .48^***^ | .23^***^ | .24^***^ | .25^***^ |  |
| 1. Need for cognition | .20^***^ | .23^***^ |  | .27^***^ | .30^***^ | .38^***^ | .10 | .11 | .17^**^ | .19^**^ |  |
| 1. Self-efficacy (EC) | .39^***^ | .29^***^ | .27^***^ |  | .39^***^ | .31^***^ | .18^**^ | .31^***^ | .3^***^4 | .16^**^ |  |
| 1. Ethical sensitivity | .51^***^ | .52^***^ | .30^***^ | .39^***^ |  | .28^***^ | .64^***^ | .36^***^ | .36^***^ | .30^***^ |  |
| 1. Conscientiousness | .30^***^ | .39^***^ | .38^***^ | .31^***^ | .28^***^ |  | .23^***^ | .07 | .17^**^ | .41^***^ |  |
| 1. Agreeableness | .38^***^ | .48^***^ | .09 | .17^**^ | .64^***^ | .22^***^ |  | .25^***^ | .16^**^ | .39^***^ |  |
| 1. Boycott intention | .46^***^ | .22^***^ | .10 | .31^***^ | .36^***^ | .06 | .24^***^ |  | .69^***^ | -.04 |  |
| 1. Negative WOM | .46^***^ | .23^***^ | .16^***^ | .34^***^ | .36^***^ | .16^**^ | .15^*^ | .69^***^ |  | -.05 |  |
| 1. Social desirability | .11 | .25^***^ | .18^***^ | .15^***^ | .30^***^ | .41^***^ | .39^***^ | -.04 | -.06 |  |  |
| 1. Marker | -.09 | -.11 | -.10 | -.01 | -.09 | -.45** | -.13^*^ | -.06 | .03 | -.38^***^ |  |

*Note*: Correlations adjusted for the smallest correaltion with the marker are reported above the diagonal. FAIRFOOD = consumers’ disposition toward fairness in food supply chains

^***^ *p* < .001; ^**^*p* < .01; ^*^ *p* < .05*;*

**3. Supplement materials for Study 4**

Table 9. Measurements used in Study 4.

| Construct | Source/  Adapted from | Item | Response format |
| --- | --- | --- | --- |
| Willingness to Purchase | Balabanis & Diamantopoulos, (2016); Giampietri et al., (2016); De Pelsmacker & Janssens, (2007); Zerbini et al., (2019) | How willing are you to purchase the following types of products^a^: | 1= Extremely unlikely;  7 = Extremely likely |
|  |  | Fair trade chocolate |  |
|  |  | Fair trade fruits (e.g. bananas, mango, avocado, etc) |  |
|  |  | Rainforest Alliance certified coffee |  |
|  |  | Fruits and vegetables from a local farmers’ market |  |
|  |  | Subscribe to an organic box scheme |  |
|  |  | Fresh products |  |
|  |  | Food with traceability information |  |
| Frequency of Engagement | Habashi et al., (2016) | Approximately, how often did you buy the following products or engage in the following activities in the last year? ^a^ | 1 = Never;  7 = More often than once a week |
|  |  | 1. Buy a certified fair trade food product |  |
|  |  | Buy a Rainforest Alliance certified food product |  |
|  |  | Visit a farmers’ market |  |
|  |  | Visit a farm shop to buy food |  |
| Experience of Positive Emotions | De Raad & Kokkonen, (2000); Snippe et al., (2018) | When I buy products that ensure fairness in food supply chain... ^a^ | 1 = Strongly disagree; 7 = Strongly agree |
|  |  | ... I feel proud |  |
|  |  | ... I feel enthusiastic |  |
|  |  | ... I feel inspired |  |
| Experience of Negative Emotions | De Raad & Kokkonen, (2000); Watson et al. (1988) | When I do not buy products that ensure fairness in food supply chain^a^ | 1 = Strongly disagree;  7 = Strongly agree |
|  |  | ... I feel guilty |  |
|  |  | ... I feel ashamed |  |
|  |  | ... I feel upset |  |
| Commitment | Eisingerich and Rubera (2010) | I feel loyal towards fair trade certified products | 1 = Strongly disagree;  7 = Strongly agree |
|  |  | Even if fair trade certified products would be more difficult to buy, I would buy them |  |
|  |  | I am willing “to go the extra mile” to purchase fair trade certified products |  |
|  |  | I will not buy fair trade certified products in the future (R) |  |
|  |  | I am committed to purchasing fair trade certified products |  |
| *Note*: ^a^ = item stem. R = reverse coded item. Social media self-control failure was used as a marker variable as in Study 2. | | | |

Table 10. Model comparison results from the CFA marker variable analysis (Study 4).

| Model | χ^2^ | *df* | CFI | Δχ^2^(Δdf) | Model Comparison |
| --- | --- | --- | --- | --- | --- |
| Italy | | | | | |
| CFA | 1239.81 | 640 | .922 | — | — |
| Baseline | 1259.15 | 652 | .921 | — | — |
| Method-C | 1259.24 | 651 | .920 | 0.15 (1) ^n.s.^ | vs. Baseline |
| Method-U | 1212.87 | 617 | .922 | 45.44 (34) ^n.s.^ | vs. Method-C |
| U.K. | | | | | |
| CFA | 1155.95 | 569 | .923 | — | — |
| Baseline | 1168.50 | 581 | .923 | — | — |
| Method-C | 1164.45 | 580 | .924 | 3.71 (1) ^n.s.^ | vs. Baseline |
| Method-U | 1112.30 | 548 | .926 | 51.57 (32)^*^ | vs. Method-C |
| Method-R | 1109.16 | 563 | .929 | 0.23 (15) ^n.s.^ | vs. Method-U |
| Note: Scaled χ2 and Δχ2 are reported. df = degrees of freedom.  ^**^*p* < .001; ^*^*p* < .05; ^n.s.^ = non-significant (*p* > .05) | | | | | |

Table 11. Results of invariance tests (Study 4).

|  | Models | χ^2^ (*df*) | Δχ^2^(Δ*df*) | CFI | RMSEA | SRMR | ΔCFI | ΔRMSEA | ΔSRMR |
| --- | --- | --- | --- | --- | --- | --- | --- | --- | --- |
| **Italy vs. U.K.** |  |  |  |  |  |  |  |  |  |
| M1: Configural invariance | — | 255.44(146) | — | .976 | .046 | .034 | — | — | — |
| M2: Metric invariance of the first-order factors | M2: M1 | 263.79(156) | 8.72 (10)^n.s.^ | .976 | .044 | .038 | .00 | .002 | .004 |
| M3: Metric invariance of the first- and second- order factors | M3: M2 | 260.59 (159) | 0.26 (3)^n.s.^ | .978 | .043 | .039 | .002 | .001 | .001 |
| M4: Scalar invariance of the first-order factors | M4: M3 | 293.78 (169) | 38.56(10)^**^ | .973 | .046 | .042 | .005 | .003 | .003 |
| M5: Scalar invariance of the first- and second- order factors | M5: M4 | 325.20 (172) | 41.25 (3)^**^ | .966 | .050 | .048 | .007 | .004 | .006 |
| **Italy: Male vs. Female** |  |  |  |  |  |  |  |  |  |
| M1: Configural invariance | — | 257.66 (146) | — | .959 | .067 | .039 | — | — | — |
| M2: Metric invariance of the first-order factors | M2: M1 | 262.1 (156) | 7.51(10)^n.s.^ | .961 | .063 | .050 | .002 | .004 | .011 |
| M3: Metric invariance of the first- and second- order factors | M3: M2 | 262.88 (159) | 1.29 (3)^n.s.^ | .962 | .062 | .053 | .001 | .001 | .003 |
| M4: Scalar invariance of the first-order factors | M4: M3 | 271.23 (169) | 5.84 (10)^n.s.^ | .963 | .059 | .053 | .001 | .003 | .00 |
| M5: Scalar invariance of the first- and second- order factors | M5: M4 | 273.37 (172) | 1.28 (3)^n.s.^ | .963 | .059 | .053 | .00 | .00 | .00 |
| **U.K.: Male vs. Female** |  |  |  |  |  |  |  |  |  |
| M1: Configural invariance | — | 196.00 (146) | — | .979 | .044 | .042 | — | — | — |
| M2: Metric invariance of the first-order factors | M2: M1 | 199.30 (156) | 3.19 (10)^n.s.^ | .981 | .040 | .044 | .002 | .004 | .002 |
| M3: Metric invariance of the first- and second- order factors | M3: M2 | 202.46 (159) | 3.42 (3)^n.s.^ | .981 | .040 | .057 | .00 | .00 | .013 |
| M4: Scalar invariance of the first-order factors | M4: M3 | 220.93 (169) | 20.08 (10)^*^ | .978 | .042 | .058 | .003 | .002 | .001 |
| M5: Scalar invariance of the first- and second- order factors | M5: M4 | 226.80 (172) | 6.60 (3)^n.s.^ | .977 | .043 | .059 | .001 | .001 | .001 |
| *Note*: Scaled χ^2^ and Δχ^2^ are reported. *df* = degrees of freedom.  ^*^ *p* < .05*;* ^**^*p* < .001; ^n.s.^ = non-significant (*p* > .05). | | | | | | | | | |

Table 12. Results of PLSpredict procedure (Study 4).

|  |  | Italy | | |  | U.K. | | |
| --- | --- | --- | --- | --- | --- | --- | --- | --- |
|  | Indicators | Q²predict | PLS-SEM (RMSE) | LM (RMSE) |  | Q²predict | PLS-SEM (RMSE) | LM (RMSE) |
| 1 | CMT 1 | 0.219 | 1.25 | 1.279 |  | 0.195 | 1.311 | 1.292 |
| 2 | CMT 2 | 0.249 | 1.234 | 1.207 |  | 0.219 | 1.212 | 1.206 |
| 3 | CMT 3 | 0.168 | 1.315 | 1.327 |  | 0.229 | 1.244 | 1.178 |
| 4 | CMT 5 | 0.242 | 1.3 | 1.288 |  | 0.255 | 1.186 | 1.148 |
| 5 | ENE 1 | 0.138 | 1.547 | 1.556 |  | 0.092 | 1.487 | 1.509 |
| 6 | ENE 2 | 0.054 | 1.415 | 1.432 |  | 0.081 | 1.338 | 1.344 |
| 7 | ENE 3 | 0.112 | 1.429 | 1.453 |  | 0.089 | 1.199 | 1.191 |
| 8 | EPE 1 | 0.137 | 1.199 | 1.225 |  | 0.113 | 1.236 | 1.222 |
| 9 | EPE 2 | 0.129 | 1.294 | 1.31 |  | 0.157 | 1.202 | 1.2 |
| 10 | EPE 3 | 0.128 | 1.252 | 1.271 |  | 0.105 | 1.303 | 1.248 |
| 11 | FRE 1 | 0.111 | 1.491 | 1.486 |  | 0.135 | 1.488 | 1.464 |
| 12 | FRE 2 | 0.08 | 1.561 | 1.535 |  | 0.128 | 1.578 | 1.571 |
| 13 | FRE 3 | 0.052 | 1.619 | 1.596 |  | 0.03 | 1.293 | 1.26 |
| 14 | FRE 4 | 0.034 | 1.752 | 1.691 |  | 0.004 | 1.341 | 1.325 |
| 15 | WTP 1 | 0.079 | 1.431 | 1.442 |  | 0.167 | 1.486 | 1.515 |
| 16 | WTP 2 | 0.121 | 1.327 | 1.305 |  | 0.16 | 1.217 | 1.255 |
| 17 | WTP 3 | 0.079 | 1.653 | 1.669 |  | 0.087 | 1.703 | 1.673 |
| 18 | WTP 4 | 0.208 | 1.182 | 1.212 |  | — | — | — |
| 19 | WTP 5 | 0.173 | 1.307 | 1.305 |  | 0.043 | 1.771 | 1.748 |
| 20 | WTP 6 | 0.137 | 0.85 | 0.884 |  | — | — | — |
| 21 | WTP 7 | 0.352 | 0.992 | 0.988 |  | 0.167 | 1.241 | 1.245 |

**References**

Adams, J. S. (1965). Inequity in social exchange. *Advances in Experimental Social Psychology, 2*, 267-299. <https://doi.org/10.1016/S0065-2601(08)60108-2>

Alvaro, C. (2017). Ethical veganism, virtue, and greatness of the soul. *Journal of Agricultural and Environmental Ethics, 30*(6), 765-781. <https://doi.org/10.1007/s10806-017-9698-z>.

Aprile, M. C., Caputo, V., & Nayga Jr., R. M. (2012). Consumers’ valuation of food quality labels: The case of the. *European Review of Agricultural Economics, 36*, 158-165. <https://doi.org/10.1111/j.1470-6431.2011.01092.x>.

Aquino, K., & Reed, A. (2002). The self-importance of moral identity. *Journal of Personality and Social Psychology*, *83*(6), 1423–1440. https://doi.org/10.1037/0022-3514.83.6.1423

Arulingam, I., Brady, G., Chaya, M., Conti, M., Kgomotso, P. K., Korzenszky, A., Njie, D., Schroth, G., & Suhardiman, D. (2022). Small-scale producers in sustainable agrifood systems transformation. Rome, FAO. <https://doi.org/10.4060/cc0821en>

Balabanis, G., & Diamantopoulos, A. (2016). Consumer xenocentrism as determinant of foreign product preference: A system justification perspective. *Journal of International Marketing, 24*(3), 58-77. <https://doi.org/10.1509/jim.15.0138>

Bartels, J., & Onwezen, M. C. (2014). Consumers’ willingness to buy products with environmental and ethical claims: The roles of social representations and social identity. *International Journal of Consumer Studies, 38*, 82-89. <https://doi.org/10.1111/ijcs.12067>.

Beck V, Ladwig B. (2021) Ethical consumerism: Veganism. WIREs Clim Change. <https://doi.org/10.1002/wcc.689>

Bolton, L. E., Warlop, L., & Alba, J. W. (2003). Consumer perceptions of price (un)fairness. *Journal of Consumer Research*, 29(4), 474–491. <https://doi.org/10.1086/346244>

Briggeman, B. C., & Lusk, J. L. (2011). Preferences for fairness and equity in the food system. *European Review of Agricultural Economics, 38*(1), 1-29. <https://doi.org/10.1093/erae/jbq033>

Brenton, S. (2018). (Political) Consumers and certification schemes: The ethics of global production and trade. *Journal of Agricultural and Environmental Ethics, 31*(6), 755-584. <https://doi.org/10.1007/s10806-018-9754-3>.

Busch, G., & Spiller, A. (2016). Farmer share and fair distribution in food chains from a consumer’s perspective. *Journal of Economic Psychology, 55*, 149-158. <https://doi.org/10.1016/j.joep.2016.03.007>.

Cacioppo, J. T., Petty, R. E., & Feng Kao, C. (1984). The efficient assessment of need for cognition. *Journal of Personality Assessment, 48*(3), 306-307. <https://doi.org/10.1207/s15327752jpa4803_13>

Chang, J. B., & Lusk, J. L. (2009). Fairness and food choice. *Food Policy, 34*(6), 483-491. <https://doi.org/10.1016/j.foodpol.2009.08.002>.

Cho, S.-H., Fang, X., Tayur, S., & Xu, Y. (2019). Combating child labor: Incentives and information disclosure in global supply chains. *Manufacturing & Service Operations Management, 21*(3), 692-711. <https://doi.org/10.1287/msom.2018.0733>

Czeczotko, M., Hanna, G., & Laskowski, W. (2021). Towards sustainable private labels in an autonomous community during COVID-19 — Analysis of consumer behavior and perception on the example of Tenerife. *Sustainability, 13*(13), 7467. <https://doi.org/10.3390/su13137467>

De Olde, E. M., & Valentinov, V. (2019). The moral complexity of agriculture: A challenge for corporate social responsibility. *Journal of Agricultural and Environmental Ethics, 32*(3), 413-430. <https://doi.org/10.1007/s10806-019-09782-3>.

De Pelsmaeker, S., Schouteten, J. J., Lagast, S., Dewettinck, K., & Gellynck, X. (2017). Is taste the key driver for consumer preference? A conjoint analysis study. *Food Quality and Preference, 62*, 323-331. <https://doi.org/10.1016/j.foodqual.2017.02.018>

De Pelsmacker, P., Janssens, W., & Mielants, C. (2005). Consumer values and fair-trade beliefs, attitudes and buying behaviour. *International Review on Public and Nonprofit Marketing*. <https://doi.org/10.1007/BF02893257>

De Pelsmacker, P., & Janssens, W. A. (2007). Model for fair trade buying behaviour: The role of perceived quantity and quality of information and of product-specific attitudes. *Journal of Business Ethics, 75*, 361-380. <https://doi.org/10.1007/s10551-006-9259-2>

De Raad, B., & Kokkonen, M. (2000). Traits and emotions: a review of their structure and management. European Journal of Personality, 14(5), 477-496. [https://doi.org/10.1002/1099-0984(200009/10)14:5<477::AID-PER396>3.0.CO;2-I](https://doi.org/10.1002/1099-0984(200009/10)14:5%3C477::AID-PER396%3E3.0.CO;2-I)

Diller, H. (2008). Price fairness. *Journal of Product and Brand Management, 17*(5), 353-355. <https://doi.org/10.1108/10610420810896103>

Du, J., van Koningsbruggen, G. M., & Kerkhof, P. (2018). A brief measure of social media self-control failure. *Computers in Human Behavior, 84*, 68-75. <https://doi.org/10.1016/j.chb.2018.02.002>

Eisingerich, A. B., & Rubera, G. (2010). Drivers of brand commitment: A cross-national investigation. *Journal of International Marketing, 18*(2), 64-79. <https://doi.org/10.1509/jimk.18.2.64>

European Parliament Directive (EU) 2019/633 on unfair trading practices in business-to-business relationships in the agricultural and food supply chain. *Official Journal of the European Union*. Retrieved from <http://data.consilium.europa.eu/doc/document/PE-4-2019-INIT/en/pdf>.

European Parliament (2011). Regulation (EU) no 1169/2011 of the European Parliament and of the Council of 25 October 2011 on the provision of food information to consumers.

European Parliament (2021). European Parliament resolution of 16 September 2021 on fair working conditions, rights and social protection for platform workers – new forms of employment linked to digital development (2019/2186(INI))

Fairtrade International. (2022). Key issues. Retrieved July 19, 2022, from <https://www.fairtrade.net/issue>.

FAO (2021a). FAO and ACPHA. 2021. Reducing child labour in agriculture in humanitarian contexts. Background paper. Rome, FAO. <https://doi.org/10.4060/cb7362en>

FAO. (2021b). FAO. 2021. The State of Food and Agriculture 2021. Making agrifood systems more resilient to shocks and stresses. Rome, FAO. https://doi.org/10.4060/cb4476en

FAO. (2023). Food Loss and Food Waste, <https://www.fao.org/policy-support/policy-themes/food-loss-food-waste/en/>

Faruk, A. (2018). Price Fairness, Satisfaction , and Trust as Antecedents of Purchase Intentions towards Organic Food. *Journal of Consuemer Behavior*, 17, 141–48. https://doi.org/10.1002/cb.1697.

Food Ethics Council. (2020). On the road to food justice - A framework for a fair food future. Retrieved from <https://www.foodethicscouncil.org/wp-content/uploads/2020/09/On-the-road-to-food-justice_FINAL.pdf>

Gielissen, R., & Graafland, J. (2009). Concepts of price fairness: Empirical research into the Dutch coffee market. *Business Ethics: A European Review, 18*(2), 165-178. <https://doi.org/10.1111/j.1467-8608.2009.01555.x>

Giampietri, E., Finco, A., Verneau, F., & Del Giudice, T. (2016). The short food supply chains’ phenomenon: A multidisciplinary approach to explore consumer behaviour and preferences. Retrieved from <https://iris.univpm.it/retrieve/handle/11566/245486/41772/tesi_giampietri.pdf#page=75>

Golob, U., & Kronegger, L. (2019). Environmental consciousness of European consumers: A segmentation-based study. *Journal of Cleaner Production, 221*, 1-9. <https://doi.org/10.1016/j.jclepro.2019.02.197>

Greenberg, J. (1990). Organizational justice: Yesterday, today, and tomorrow. *Journal of Management, 16*(2), 399-432.

Grumett, D. (2019). Aristotle’s ethics and farm animal welfare. *Journal of Agricultural and Environmental Ethics, 32*(2), 321-333. <https://doi.org/10.1007/s10806-019-09776-1>.

Gudbrandsdottir, I. Y., Olafsdottir, G., Oddsson, G. V., Stefansson, H., & Bogason, S. G. (2021). Operationalization of interorganizational fairness in food systems: From a social construct to quantitative indicators. *Agriculture, 11*(1), 36. <https://doi.org/10.3390/agriculture11010036>

Habashi, M. M., Graziano, W. G., & Hoover, A. E. (2016). Searching for the prosocial personality: A big five approach to linking personality and prosocial behavior. *Personality and Social Psychology Bulletin, 42*(9), 1177-1192. <https://doi.org/10.1177/0146167216652859>

Hoang, V. (2021). Modern short food supply chain, good agricultural practices, and sustainability: A conceptual framework and case study. *Agronomy, 11*(12), 2408. <https://doi.org/10.3390/agronomy11122408>

Höglund, A. T. (2020). What shall we eat? An ethical framework for well-grounded food choices. *Journal of Agricultural and Environmental Ethics, 33*(2), 283-297. <https://doi.org/10.1007/s10806-020-09821-4>.

IFOAM. Fairness and transparency Strengthening the position of more vulnerable actors in the food supply chain https://www.organicseurope.bio/what-we-do/fairness-transparency/

Jeong, H., Suri, R., Ye, H., Bhatt, S. (2021). When should retailers increase prices during a crisis? A longitudinal inquiry during the COVID-19 pandemic. *Journal of Consumer Behaviour, 20*, 1269-1276. <https://doi.org/10.1002/cb.1934>.

Kilbourne, W., & Pickett, G. (2008). How materialism affects environmental beliefs, concern, and environmentally responsible behavior. *Journal of Business Research, 61*(9), 885-893. <https://doi.org/10.1016/j.jbusres.2007.09.016>

Klein, J. G., Smith, N. C., & John, A. (2004). Why we boycott: Consumer motivations for boycott participation. *Journal of Marketing, 68*(3), 92-109. <https://doi.org/10.1509/jmkg.68.3.92.34770>

Korzen, S., Sandøe, P., and Lassen, J. (2011). Pure meat—public perceptions of risk reduction strategies in meat production. *Food Policy*. 36, 158–165. doi: 10.1016/j.foodpol.2010.10.005

Lasarov, W., Hoffmann, S., & Orth, U. (2023). Vanishing boycott impetus: Why and how consumer participation in a boycott decreases over time. *Journal of Business Ethics, 182*(4), 1129-1154. <https://doi.org/10.1007/s10551-021-04997-9>

Maples-Keller, J. L., Williamson, R. L., Sleep, C. E., Carter, N. T., Campbell, W. K., & Miller, J. D. (2019). Using item response theory to develop a 60-item representation of the NEO PI–R using the International Personality Item Pool: Development of the IPIP–NEO–60. *Journal of Personality Assessment, 101*(1), 4-15. <https://doi.org/10.1080/00223891.2017.1381968>

Nawroth, C., Langbein, J., Coulon, M., Gabor, V., Oesterwind, S., Benz-Schwarzburg, J., & Von Borell, E. (2019). Farm animal cognition—linking behavior, welfare and ethics. *Frontiers in Veterinary Science, 6*(February), 1-16. <https://doi.org/10.3389/fvets.2019.00024>

Nguyen, B., & Klaus, P. P. (2013). Retail fairness: Exploring consumer perceptions of fairness towards retailers' marketing tactics. *Journal of Retailing and Consumer Services, 20*, 311-324. <https://doi.org/10.1016/j.jretconser.2013.02.001>

Peano, C., Merlino, V. M., Sottile, F., Borra, D., & Massaglia, S. (2019). Sustainability for food consumers: Which perception? *Sustainability, 11*(21), 5955. <https://doi.org/10.3390/su11215955>

Reis, G. G., Molento, C. F. M., & Souza, A. P. O. (2021). Governance and standardization in fish value chains: Do they take care of key animal welfare issues? *Journal of Agricultural and Environmental Ethics, 34*, 30. <https://doi.org/10.1007/s10806-021-09870-3>

Rozin, P., Spranca, M., Krieger, Z., Neuhaus, R., Surillo, D., Swerdlin, A., ... (2004). Preference for natural: Instrumental and ideational/moral motivations, and the contrast between foods and medicines. *Appetite, 43*, 147-154. <https://doi.org/10.1016/j.appet.2004.03.005>

Sacchi, G. (2018). The ethics and politics of food purchasing choices in Italian consumers’ collective action. *Journal of Agricultural and Environmental Ethics, 31*(1), 73-91. <https://doi.org/10.1007/s10806-018-9710-2>

Samoggia, A., Grillini, G., & Del Prete, M. (2021). Price fairness of processed tomato agro-food chain: The Italian consumers' perception perspective. *Foods, 10*(5), 984. <https://doi.org/10.3390/foods10050984>

Shaw, D., Grehan, E., Shiu, E., Hassan, L., & Thomson, J. (2005). An exploration of values in ethical consumer decision making. *Journal of Consumer Behaviour, 4*, 185-200. <https://doi.org/10.1002/cb.3>

Shih-Tse, W. E., & Chen, Y.-C. (2019). Effects of perceived justice of fair trade organizations on consumers’ purchase intention toward fair trade products. *Journal of Retailing and Consumer Services, 50*, 66-72. <https://doi.org/10.1016/j.jretconser.2019.05.004>

Smith, V., Møgelvang-Hansen, P., & Hyldig, G. (2010). Spin versus fair speak in food labelling: A matter of taste? *Food Quality and Preference, 21*(8), 1016-1025. <https://doi.org/10.1016/j.foodqual.2010.05.016>

Snippe, E., Jeronimus, B. F., aan het Rot, M., Bos, E. H., de Jonge, P., & Wichers, M. (2018). The Reciprocity of Prosocial Behavior and Positive Affect in Daily Life. *Journal of personality*, *86*(2), 139-146. [https://doi.org/https://doi.org/10.1111/jopy.12299](https://doi.org/https:/doi.org/10.1111/jopy.12299)

Sudbury-Riley, L., & Kohlbacher, F. (2016). Ethically minded consumer behavior: Scale review, development, and validation. *Journal of Business Research, 69*(8), 2697-2710. <https://doi.org/10.1016/j.jbusres.2015.11.005>

Swaffield, S. R., Primdahl, J., Corry, R. C., Opdam, P., & Mcwilliam, W. (2019). Connecting business with the agricultural landscape: Business strategies for sustainable rural development. *Business Strategy and the Environment, April*, 1357-1369. <https://doi.org/10.1002/bse.2320>

Thøgersen, J. (1999). The ethical consumer. Moral norms and packaging choice. *Journal of Consumer Policy, 22*, 439-460. <https://doi.org/10.1023/A:1006225711603>

Tian, L., Tse, C. H., Xiang, X., Li, Y., & Pan, Y. (2021). Social movements and international business activities of firms. *Journal of International Business Studies, 52*(6), 1200-1214. <https://doi.org/10.1057/s41267-021-00424-3>

Toti, J.-F., Diallo, M. F., & Huaman-Ramirez, R. (2021). Ethical sensitivity in consumers’ decision-making: The mediating and moderating role of internal locus of control. *Journal of Business Research, 131*, 168-182. <https://doi.org/10.1016/j.jbusres.2021.03.045>

Toti, J.-F., & Moulins, J.-L. (2017). Ethical sensitivity: Conceptualization and new scale development. *Recherche et Applications En Marketing (English Edition), 32*(3), 6-27. <https://doi.org/10.1177/2051570717716562>

Vésteinsdóttir, V., Reips, U.-D., Joinson, A., & Thorsdottir, F. (2017). An item level evaluation of the Marlowe-Crowne Social Desirability Scale using item response theory on Icelandic Internet panel data and cognitive interviews. *Personality and Individual Differences, 107*, 164-173. <https://doi.org/10.1016/j.paid.2016.11.023>

Watson, D., Clark, L. A., & Tellegen, A. (1988). Development and validation of brief measures of positive and negative affect: the PANAS scales. *Journal of Personality and Social Psychology*, 54(6), 1063.

Winterstein, J., André Habisch, A. (2019). Is local the new organic? Empirical evidence from German regions. *British Food Journal*. <https://doi.org/10.1108/BFJ-06-2020-0517>

Wood, M. S., & Karau, S. J. (2009). Preserving employee dignity during the termination interview: An empirical examination. *Journal of Business Ethics, 86*(4), 519-534. <https://doi.org/10.1007/s10551-008-9862-5>

Zarantonello, L., Romani, S., Grappi, S., & Bagozzi, R. P. (2016). Brand hate. *Journal of Product & Brand Management, 25*(1), 11-25, doi:10.1108/JPBM-01-2015-0799.

Zepeda, L., Sirieix, L., Pizarro, A., Corderre, F., & Rodier, F. (2013). A conceptual framework for analyzing consumers’ food label preferences: An exploratory study of sustainability labels in France, Quebec, Spain, and the US. *International Journal of Consumer Studies, 37*(2008), 605-616. <https://doi.org/10.1111/ijcs.12041>

Zerbini, C., Vergura, D. T., & Latusi, S. (2019). A new model to predict consumers' willingness to buy fair-trade products. *Food Research International, 122*, 167-173. <https://doi.org/10.1016/j.foodres.2019.04.008>

Zimmerer, K. S., De Haan, S., Jones, A. D., Creed-Kanashiro, H., Tello, M., Carrasco, M., Meza, K., ... (2019). The biodiversity of food and agriculture (Agrobiodiversity) in the Anthropocene: Research advances and conceptual framework. *Biochemical Pharmacology, 25*, 100192. <https://doi.org/10.1016/j.ancene.2019.100192>
